# Product Engagement Detection Using Multi-Camera 3D Skeleton Reconstruction and Gaze Estimation

**DOI:** 10.3390/s25103031

**Published:** 2025-05-11

**Authors:** Matus Tanonwong, Yu Zhu, Naoya Chiba, Koichi Hashimoto

**Affiliations:** 1 Graduate School of Information Sciences, Tohoku University, Aoba-ku, Sendai 980-8579, Japan; tanonwong.matus.q5@dc.tohoku.ac.jp (M.T.); zhu.yu.s3@dc.tohoku.ac.jp (Y.Z.); 2 Graduate School of Information Science and Technology, Osaka University, 1-32 Machikaneyama, Toyonaka, Osaka 560-0043, Japan; chiba@nchiba.net

**Keywords:** product engagement detection, retail analytics, gaze estimation, 2D/3D imaging, multi-camera system, computer vision

## Abstract

Product engagement detection in retail environments is critical for understanding customer preferences through nonverbal cues such as gaze and hand movements. This study presents a system leveraging a 360-degree top-view fisheye camera combined with two perspective cameras, the only sensors required for deployment, effectively capturing subtle interactions even under occlusion or distant camera setups. Unlike conventional image-based gaze estimation methods that are sensitive to background variations and require capturing a person’s full appearance, raising privacy concerns, our approach utilizes a novel Transformer-based encoder operating directly on 3D skeletal keypoints. This innovation significantly reduces privacy risks by avoiding personal appearance data and benefits from ongoing advancements in accurate skeleton estimation techniques. Experimental evaluation in a simulated retail environment demonstrates that our method effectively identifies critical gaze-object and hand-object interactions, reliably detecting customer engagement prior to product selection. Despite yielding slightly higher mean angular errors in gaze estimation compared to a recent image-based method, the Transformer-based model achieves comparable performance in gaze-object detection. Its robustness, generalizability, and inherent privacy preservation make it particularly suitable for deployment in practical retail scenarios such as convenience stores, supermarkets, and shopping malls, highlighting its superiority in real-world applicability.

## 1. Introduction

Understanding customer behavior in retail environments is crucial for optimizing product placement, merchandising, and marketing strategies. Nonverbal cues, such as where a customer directs their gaze and how they physically interact with products, offer valuable insights into underlying preferences and decision-making processes. These observations help reveal which items attract attention, the order in which they are examined, and which interactions lead to purchase decisions. Such behavioral data can inform layout design, targeted promotions, inventory management, and even the personalization of shopping experiences.

However, detecting these nonverbal cues in real-world environments remains a significant challenge. Conventional image-based methods for gaze and hand detection are highly sensitive to background clutter, occlusions, limited camera viewpoints, and variable lighting conditions. Furthermore, they often require high-quality facial or hand images and raise privacy concerns due to reliance on visual appearance. These factors reduce the reliability and generalizability of such systems in unconstrained retail environments like supermarkets or convenience stores.

In this paper, we aim to develop a robust, privacy-preserving framework for detecting product engagement, defined as both hand-object and gaze-object interactions, under real-world constraints. Our system reconstructs 3D human skeletons from synchronized multi-view camera data and uses these skeletal representations for both gaze estimation and hand-object engagement detection. By avoiding direct use of appearance-based features, our method ensures greater robustness across scenes and preserves customer anonymity.

**Figure 1:**
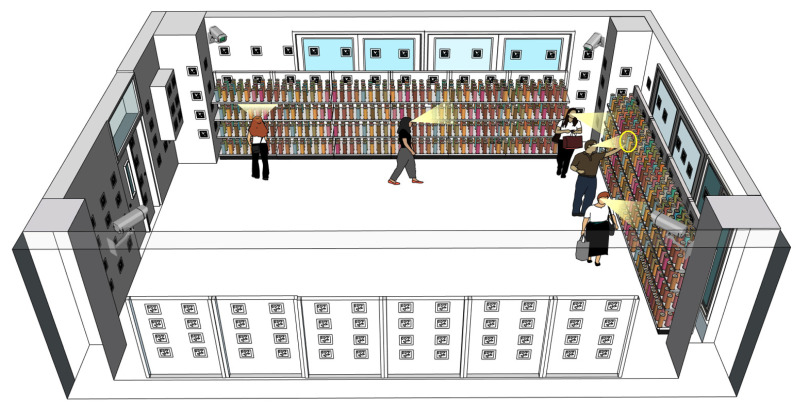
The 3D layout of our convenience store environment: hand and gaze information indicate product engagement and customer interests.

Product engagement encompasses both physical contact with products and visual attention directed toward them. While hand interactions typically indicate a strong level of intent, such as picking up, holding, or examining a product, gaze interactions reflect earlier stages of interest, including initial attraction, visual comparison, and indecision. The gaze can also precede or follow hand engagement, offering a temporal dimension that reveals how attention evolves over time. By analyzing these modalities together, we can uncover detailed behavioral patterns such as hesitations before picking a product, prolonged attention without physical interaction, or quick decisions driven by immediate engagement. This combination offers a more complete picture of customer behavior on the shelf, including the cognitive and physical components of decision-making. The fusion of hand and gaze signals, as illustrated in Figure 1, enables retailers to optimize product placements, identify attention hotspots, and design interventions, such as signage or dynamic pricing, which convert interest into action. Ultimately, such insights can support data-driven strategies to enhance customer experience, increase sales conversion, and improve overall store performance.

To achieve this, our system integrates data from a 360-degree top-view fisheye camera and two perspective cameras. This multi-view configuration overcomes limitations related to occlusion and restricted fields of view, enabling accurate 3D keypoint reconstruction even under challenging conditions. These reconstructed skeletons form the basis for both hand-object interaction analysis and 3D gaze estimation using a Transformer-based encoder. By operating on 3D keypoints rather than raw image appearance, our method remains robust to visual variation and eliminates privacy concerns associated with conventional image-based approaches.

Our work provides the following key contributions:

- We propose an innovative system for product engagement detection that integrates multi-camera 3D skeleton reconstruction with a gaze estimation framework, providing a comprehensive view of customer behavior by capturing both physical interactions and visual attention.
- We introduce a novel Transformer-based skeleton encoder for gaze estimation that leverages full-body skeletal cues, enhancing robustness against background variations and lighting changes. Unlike image-based methods that rely on pixel data and are sensitive to environmental fluctuations, our approach uses raw 3D skeleton keypoints to filter out extraneous details, resulting in a more generalizable gaze estimation.
- Our evaluations confirm that product engagement was successfully detected. This achievement is enabled by our multi-camera approach, which is supported by robust calibration and the use of a Kalman filter, by accurate 3D skeleton reconstruction in the hand-object pipeline, and by gaze estimation in the gaze-object pipeline. Together, these techniques provide refined insights into customer behavior.

## 2. Related Work

Understanding customer product engagement in physical retail settings has driven extensive research in computer vision. Three major research streams have emerged, namely, (i) gaze-object detection, (ii) hand-object detection, and (iii) 3D human pose reconstruction. While gaze and hand interactions provide complementary cues about attention and intent, both streams are often challenged by occlusion, limited viewpoints, and the lack of reliable depth information in 2D imagery. To overcome these issues, 3D reconstruction methods, whether through multi-view triangulation or monocular lifting from 2D keypoints, have increasingly been adopted to enhance spatial reasoning and improve robustness in complex environments.

### 2.1. Gaze-Object Detection

Early research tackled the problem of tracking physical browsing using wearable sensors. Ref. [1] leveraged first-person vision and inertial sensing to track customer navigation, identifying regions of interest by analyzing head orientation and even inferring product layouts via an auto layout technique. In a similar vein, Ref. [2] proposed a deep neural network-based gaze-following approach to identify the object of interest, although its applicability is limited to close-up views.

Real-time gaze estimation in retail has further evolved with studies such as [3], which integrates CNN-based eye tracking with a weighted bounding box selection strategy, offering enhanced accuracy in detecting retail products in first-person views. In contrast, Ref. [4] utilized conventional CCTV footage to generate engagement heatmaps by tracking head orientation, despite its reliance on the head’s pose as a proxy for gaze direction.

Recognizing the need to bridge pixel-level annotations with object-level predictions, Ref. [5] introduced the task of gaze object prediction and provided a dedicated dataset. Building on this, Ref. [6] employed a specific-general-specific mechanism to extract features from both the scene and head image, although its performance depends on close-up views. Similarly, Ref. [7] leveraged Transformer architectures to model long-range relationships between the head and the gaze object, but it, too, requires the camera to be sufficiently close to the shelves.

Complementary strategies have been explored to refine object boundaries. Ref. [8] employed a vision foundation model (SAM) to obtain high-quality masks for gaze estimation, yet it necessitates box-level supervision, limiting its broader applicability. In addition, Ref. [9] presents an end-to-end framework that predicts multiple head–target pairs, although it is constrained by a fixed cap on instances and remains effective primarily for close-up views.

Beyond pure gaze estimation, integrated systems have been proposed. Ref. [10] fused object detection, gaze estimation, and hand gesture recognition to control IoT devices, while Ref. [11] utilized Transformer-based architectures to generate comprehensive gaze analyses that include target areas and object classes. Moreover, Ref. [12] introduced GazeSeg, a method that performs coarse-to-fine segmentation of gaze targets, again, a technique that requires clear, high-resolution imagery. Complementing these, Ref. [13] demonstrated that integrating human gaze cues with object detection can enhance scene understanding by emphasizing semantically meaningful regions. Finally, Ref. [14] applied deep learning to surveillance footage to infer customer interest via head orientation, highlighting the limitation of relying solely on estimated head poses.

However, many of these integrated systems share common limitations. For example, they often require high-quality or close-range visual data, rely heavily on facial features or appearance cues, and lack robustness in cluttered, occluded, or low-resolution environments. Moreover, few systems address privacy concerns, which is a critical consideration in commercial or public settings. These limitations highlight the need for a more generalizable and privacy-preserving framework that leverages structured representations, such as 3D human skeletons, as explored in our work.

### 2.2. Hand-Object Detection

In parallel to gaze analysis, numerous studies have focused on detecting hand-object interactions to better understand in-store customer behavior. In early work, Ref. [15] introduced the concept of “action-objects” and used a two-stream network to combine RGB appearance with 3D spatial cues, however, it was limited to first-person image views. Likewise, Ref. [16] used long-term egomotion cues to pinpoint moments of engagement from wearable cameras, and Ref. [17] achieved real-time 6D pose estimation through dense pixel labeling, both of which are confined to egocentric scenarios.

Graph-based approaches have also been explored. Ref. [18] utilized adaptive graph convolutional networks to jointly predict 2D and 3D poses from close-up views, while Ref. [19] proposed a learning-free fitting method for the 3D reconstruction of hand-object interactions, again assuming high-quality, proximal imagery.

Retail-specific applications extend these approaches to top-view surveillance. For instance, Ref. [20] developed a low-cost system that detects customer-merchandise interactions from close-up top-view images, although it suffers from a lack of depth information. Similarly, Ref. [21] tracked customer hand movements and hand status using tiny-YOLOv3 and MobileNetV2, but its reliance on 2D overlap computations limits its robustness in the absence of depth cues.

More comprehensive systems have been proposed as well. Ref. [22] presented an end-to-end framework that synchronizes multiple cameras to track products and recognize purchasing behavior, however, its requirement for multiple cameras per shelf can hinder scalability. In a related approach, Ref. [23] implemented a smart trolley system using YOLOv7, although it only provides information on items added to or removed from the cart and does not capture other forms of product engagement.

Further refinement of hand-object engagement is found in [24], which combined pose estimation via BlazePose with object detection to track shopping behaviors in real time. Yet again, the method is designed for close-up top-view images, lacking depth analysis. Ref. [25] advanced the field by incorporating RGB-D data for classifying customer interactions, though it remains limited to controlled, close-range settings. In contrast, Ref. [26] focused solely on object detection within vending environments, again constrained by the lack of depth information.

Recognizing the variability in product engagement activities, hierarchical models have been proposed. Ref. [27] organized customer actions into multiple levels of abstraction to adapt to dynamic store conditions, while Refs. [28,29] both leveraged primitive-based approaches to flexibly define customer behaviors; however, these approaches are still predominantly reliant on close-up top-view imagery with limited depth cues.

Systems for inventory management have also been addressed. Ref. [30] utilized multiple cameras and ROI-based detection to improve recognition in occlusion-prone environments, and Ref. [31] presented an efficient CNN framework that reduces computational load. Both methods, however, require dense camera installations to achieve acceptable performance. Finally, Ref. [32] focused on reliable real-time identity verification to ensure secure access in unmanned stores, yet its experimental evaluation is limited by dataset variability. In a related direction, Ref. [33] introduced a self-supervised framework using context-aware image mixing to differentiate interacting objects in cluttered scenes, although its performance can be compromised by occlusion and insufficient depth information when customers are far from the camera.

Collectively, these studies illustrate a rich landscape of methodologies for extracting customer behavioral cues in retail settings. Gaze-based approaches have progressed from smart glasses and egocentric networks to Transformer-based systems and pixel-level segmentation, yet they often depend on close-up or first-person views to capture fine-grained attention details, with some methods solely relying on head pose estimation. Concurrently, hand-object detection methods, from early action-object models to hierarchical and primitive-based frameworks, demonstrate promising capabilities but are frequently limited by top-view, close-range imaging, and a lack of depth information. Moreover, several systems demand complex multi-camera setups, which can be impractical in large-scale retail environments.

### 2.3. 3D Reconstruction from 2D Skeletons

Reconstructing 3D human skeletons from 2D keypoints has become a fundamental task in vision-based behavior analysis. Two main approaches have emerged in recent literature, namely, (1) multi-view stereo systems, which triangulate 2D poses from multiple calibrated cameras, and (2) monocular deep learning methods, which predict 3D skeletons directly from 2D joint coordinates.

Multi-view stereo systems use synchronized and calibrated camera setups to estimate 3D joint locations through geometric triangulation or volumetric fusion. Early large-scale systems like the Panoptic Studio [34] demonstrated how dense camera arrays can enable accurate and markerless 3D tracking, even under significant occlusions. More recent works, such as TriPose [35] and VoxelPose [36], improved the efficiency of these systems by reducing the reliance on dense camera setups, introducing robust 2D joint association strategies, and using voxel-based 3D CNNs for fusion. One study [37] further advanced this line of work by proposing a stochastic triangulation method that generalizes across different camera configurations. These systems offer high spatial accuracy, generate outputs in real-world coordinates, and scale well to multi-person tracking in complex, cluttered environments. However, they require careful calibration and infrastructure, which can limit their practicality to more controlled settings such as studios, smart retail labs, or fixed surveillance environments.

Monocular deep learning approaches, on the other hand, aim to lift 2D poses into 3D using learned priors about human pose and motion. A foundational work, Ref. [38] introduced a simple yet strong baseline using fully connected layers to regress 3D joints from 2D keypoints. Ref. [39] extended this by incorporating weak supervision and geometric constraints, enabling training with both 2D and 3D datasets. More recent innovations have leveraged graph-based models (e.g., SemGCN [40]), temporal convolutional networks (e.g., VideoPose3D [41]), and Transformer architectures (e.g., PoseFormer [42]) to better capture pose dependencies and long-term dynamics. These methods are lightweight, real-time capable, and require minimal setup, making them attractive for scenarios where hardware constraints or deployment flexibility are priorities. Nonetheless, they are fundamentally limited by depth ambiguity, occlusion sensitivity, and lack of absolute scale factors that reduce their robustness in multi-person or cluttered environments such as retail spaces.

Given these trade-offs, our work adopts a multi-view 3D reconstruction framework based on 2D keypoint fusion. This approach leverages the flexibility and generalizability of modern 2D human pose estimators while benefiting from the geometric accuracy and occlusion robustness of triangulation-based 3D reconstruction. It enables metric localization, preserves privacy by avoiding appearance-based inputs, and supports reliable behavior analysis in dynamic retail environment.

Motivated by these challenges, our work proposes a novel approach for product engagement detection in retail environments. Our system leverages a 360-degree top-view fisheye camera combined with two perspective cameras to capture subtle hand interactions and gaze directions, even under occlusions or when the cameras are distant from subjects. In a lab-simulated retail setting, we integrated established techniques in human detection, tracking, pose estimation, and gaze estimation to reconstruct 3D human skeletons and gaze vectors from image planes. Recognizing that image-based gaze models can be sensitive to background variations, we propose a Transformer-based skeleton encoder that directly utilizes 3D keypoints for gaze estimation. By synthesizing advances from both gaze estimation and hand-object detection, our framework overcomes limitations related to view dependency, depth insufficiency, and high camera density requirements, ultimately providing a more comprehensive and generalizable solution for product engagement analysis in diverse retail settings.

## 3. Materials and Methods

### 3.1. Retail Environment Setup

Our simulated retail environment in our laboratory measures 4.476 m by 7.665 m. The cameras and sensors deployed in this study include an OptiTrack Motion Capture (MoCap) system (Motive v3.1.4; Acuity Inc., Tokyo, Japan) equipped with eight PrimeX 13 cameras (Acuity Inc., Tokyo, Japan), a DS-2CD6365G1-IVS HIK 6MP fisheye camera (HIKVISION, Hangzhou, China), SANWA CMS-V51BK web cameras (Sanwa Supply Inc., Tokyo, Japan), Pupil Core eye trackers (Pupil Labs, Berlin, Germany), and GoPro HERO11 Black cameras (GoPro Inc., San Mateo, CA, USA). While several of these sensors were used for collecting ground truth data and supporting experiments, the final deployed system relies only on the fisheye camera and two perspective cameras. We integrate these devices into the world coordinate system using the method described in our previous study [43], which enables us to estimate the positions and orientations of additional cameras and sensors relative to the fiducial markers in the environment. With 157 pre-scanned AprilTag markers, we can determine a camera’s position and pose when the markers are detected. The Pupil Core eye trackers are used to track subjects’ gaze directions, while the GoPro cameras attached to the subjects’ chests estimate their body orientations. The fisheye camera and two web cameras capture the scene.

### 3.2. 3D Reconstruction of Retail Environment

**Figure 2:**
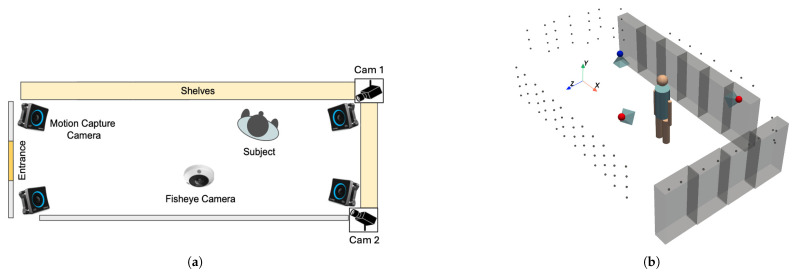
Reconstruction of the retail environment. (**a**) Top-view layout of the room showing camera locations. (**b**) Final reconstructed model of the retail space, featuring shelves positioned according to real-world measurements and integrated camera placements derived from marker detection.

After identifying the positions of 157 AprilTag markers and determining the orientations of the fisheye camera and the two web cameras (Cam1 and Cam2) as shown in Figure 2a, we constructed the retail environment complete with shelves and cameras. The positions of the shelves were determined based on the AprilTag markers affixed to them, and their dimensions were measured to reflect their actual sizes. In addition, the positions and orientations of the fisheye camera and the two web cameras were computed using the detected AprilTag markers in the environment. Finally, we reconstructed the retail environment as illustrated in Figure 2b.

### 3.3. Dataset for Evaluating Reconstructed 3D Keypoints

To verify the accuracy of our estimated camera positions and orientations, we collected a small evaluation dataset. This dataset consists of videos of three subjects, each wearing a motion capture suit with 39 markers, while performing five simple actions (picking items, returning items, reading labels, placing items in a basket, and checking wristwatches). It includes 75 videos from the motion capture system, 75 from the fisheye camera, 75 from Cam1, and 75 from Cam2, as shown in Table 1. Note that the motion capture system is used exclusively to provide ground truth data for evaluation and calibration purposes.

**Figure 3:**
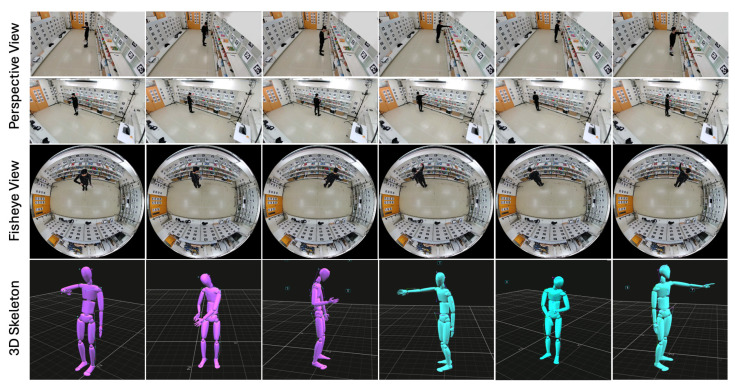
Sample frames from the evaluation dataset showing subjects in motion capture suits performing various actions. These examples illustrate the different perspectives provided by the motion capture system and the installed cameras.

The dataset comprises a total of 14,404 synchronized frames, with each frame annotated using 39 keypoints captured by the MoCap system. This results in an extensive dataset of 561,756 keypoints. The action with the highest number of synchronized frames is “Read Labels” (3826 frames, 149,214 keypoints), while “Put in Basket” contains the fewest synchronized frames (2225 frames, 86,775 keypoints). Examples of the captured dataset are illustrated in Figure 3.

**Figure 4:**
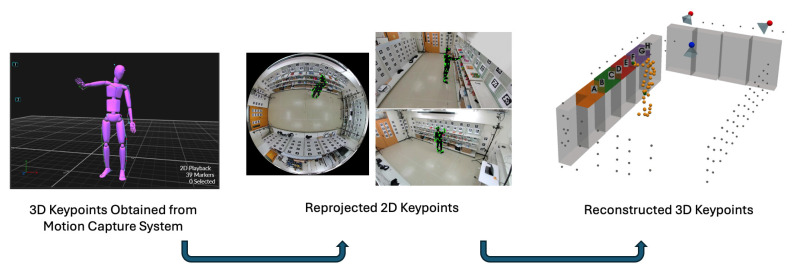
Comparison of original and reconstructed 3D keypoints. This figure displays the reprojected 2D keypoints used to reconstruct the 3D keypoints alongside the original motion capture data.

Next, we transform the 3D keypoints obtained from the motion capture system into each camera’s coordinate system to compute the reprojected 2D keypoints on the image plane. We then use these reprojected 2D keypoints to reconstruct the 3D keypoints and compare them with the original 3D keypoints from the motion capture system, as shown in Figure 4. This process verifies the accuracy of our camera integration.

#### 3.3.1. Stereo Camera View

**Figure 5:**
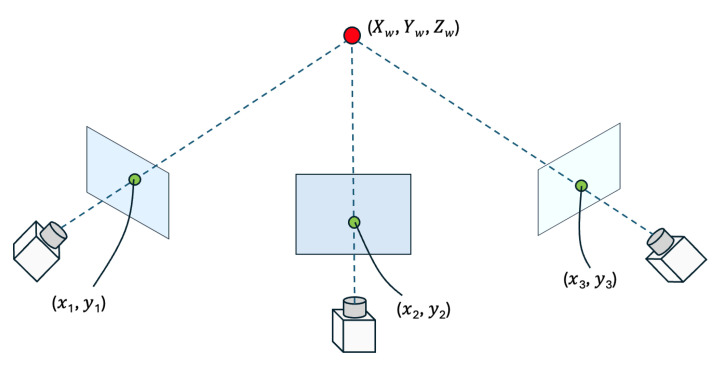
Illustration of the stereo camera setup used to reconstruct a 3D point ($X_{w},Y_{w},Z_{w}$) from its 2D projections ($x_{1},y_{1}$), ($x_{2},y_{2}$), and ($x_{3},y_{3}$) in three different cameras (a fisheye camera, Cam1, and Cam2). By applying the transformation matrices P, Q, R, we can triangulate and recover the 3D location of the point in the world coordinate system.

In a typical stereo (or multi-camera) setup, each camera is calibrated so that its intrinsic parameters (focal length, principal point, lens distortion) and extrinsic parameters (rotation and translation relative to a common reference) are known. By observing the same point in the scene from different viewpoints, we can triangulate its 3D location. Figure 5 illustrates this concept, where a single 3D point ($X_{w},Y_{w},Z_{w}$) in the world coordinate system is captured by three cameras (the fisheye camera, Cam1, and Cam2), each producing 2D pixel coordinates ($x_{1},y_{1}$), ($x_{2},y_{2}$), and ($x_{3},y_{3}$).

To relate the 3D point in the world coordinate system to its 2D projection in each camera, we define three transformation matrices:

(1) $$\begin{array}{cc} P & =\left(\begin{array}{cccc} p_{11} & p_{12} & p_{13} & p_{14}\\ p_{21} & p_{22} & p_{23} & p_{24}\\ p_{31} & p_{32} & p_{33} & p_{34} \end{array}\right),\\ Q & =\left(\begin{array}{cccc} q_{11} & q_{12} & q_{13} & q_{14}\\ q_{21} & q_{22} & q_{23} & q_{24}\\ q_{31} & q_{32} & q_{33} & q_{34} \end{array}\right),\\ R & =\left(\begin{array}{cccc} r_{11} & r_{12} & r_{13} & r_{14}\\ r_{21} & r_{22} & r_{23} & r_{24}\\ r_{31} & r_{32} & r_{33} & r_{34} \end{array}\right), \end{array}$$

where each matrix encodes the combined intrinsic and extrinsic parameters for one of the cameras. In other words, multiplying a world coordinate ($X_{w},Y_{w},Z_{w}$) by, say, P will yield the point’s coordinates in the fisheye camera’s image plane (up to a projective scale). To avoid an indeterminate scale factor, we fix the last element of each transformation matrix ($p_{34}$,$q_{34}$,$r_{34}$) to 1. This ensures that the 2D projection coordinates ($x_{i}$,$y_{i}$,1) are consistently scaled and comparable across different cameras.

(2) $$P\left(\begin{array}{c} X_{w}\\ Y_{w}\\ Z_{w} \end{array}\right)\propto \left(\begin{array}{c} x_{1}\\ y_{1}\\ 1 \end{array}\right),$$

when $p_{34}=1$, then we have the following:

(3) $$\left(\begin{array}{c} x_{1}\\ y_{1}\\ 1 \end{array}\right)∼\left(\begin{array}{cccc} p_{11} & p_{12} & p_{13} & p_{14}\\ p_{21} & p_{22} & p_{23} & p_{24}\\ p_{31} & p_{32} & p_{33} & p_{34} \end{array}\right)\left(\begin{array}{c} X_{w}\\ Y_{w}\\ Z_{w}\\ 1 \end{array}\right).$$

The same derivation applies to Q and R, so we obtain the following:

(4) $$\begin{array}{cc} p_{34}x_{1} & =X_{w}p_{11}+Y_{w}p_{12}+Z_{w}p_{13}+p_{14}-x_{1}X_{w}p_{31}-x_{1}Y_{w}p_{32}-x_{1}Z_{w}p_{33},\\ p_{34}y_{1} & =X_{w}p_{21}+Y_{w}p_{22}+Z_{w}p_{23}+p_{24}-y_{1}X_{w}p_{31}-y_{1}Y_{w}p_{32}-y_{1}Z_{w}p_{33},\\ q_{34}x_{2} & =X_{w}q_{11}+Y_{w}q_{12}+Z_{w}q_{13}+q_{14}-x_{2}X_{w}q_{31}-x_{2}Y_{w}q_{32}-x_{2}Z_{w}q_{33},\\ q_{34}y_{2} & =X_{w}q_{21}+Y_{w}q_{22}+Z_{w}q_{23}+q_{24}-y_{2}X_{w}q_{31}-y_{2}Y_{w}q_{32}-y_{2}Z_{w}q_{33},\\ r_{34}x_{3} & =X_{w}r_{11}+Y_{w}r_{12}+Z_{w}r_{13}+r_{14}-x_{3}X_{w}r_{31}-x_{3}Y_{w}r_{32}-x_{3}Z_{w}r_{33},\\ r_{34}y_{3} & =X_{w}r_{21}+Y_{w}r_{22}+Z_{w}r_{23}+r_{24}-y_{3}X_{w}r_{31}-y_{3}Y_{w}r_{32}-y_{3}Z_{w}r_{33}. \end{array}$$

Finally, we can solve for the reconstructed 3D keypoint in world coordinates ($X_{w}$,$Y_{w}$,$Z_{w}$) by applying the least-squares method with the Broyden–Fletcher–Goldfarb–Shanno (BFGS) algorithm to the following equation:

(5) $$\left(\begin{array}{ccc} p_{31}x_{1}-p_{11} & p_{32}x_{1}-p_{12} & p_{33}x_{1}-p_{13}\\ p_{31}y_{1}-p_{21} & p_{32}y_{1}-p_{22} & p_{33}y_{1}-p_{23}\\ q_{31}x_{2}-q_{11} & q_{32}x_{2}-q_{12} & q_{33}x_{2}-q_{13}\\ q_{31}y_{2}-q_{21} & q_{32}y_{2}-q_{22} & q_{33}y_{2}-q_{23}\\ r_{31}x_{3}-r_{11} & r_{32}x_{3}-r_{12} & r_{33}x_{3}-r_{13}\\ r_{31}y_{3}-r_{21} & r_{32}y_{3}-r_{22} & r_{33}y_{3}-r_{23} \end{array}\right)\left(\begin{array}{c} X_{w}\\ Y_{w}\\ Z_{w} \end{array}\right)=\left(\begin{array}{c} p_{14}-p_{34}x_{1}\\ p_{24}-p_{34}y_{1}\\ q_{14}-q_{34}x_{2}\\ q_{24}-q_{34}y_{2}\\ r_{14}-r_{34}x_{3}\\ r_{24}-r_{34}y_{3} \end{array}\right)$$

By solving these equations for each keypoint of a skeleton (e.g., each joint in a human body), we reconstruct the entire 3D pose in the world coordinate system. This method is robust to small errors in measurement or slight occlusions because using multiple camera views provides redundant information. Consequently, the reconstructed 3D keypoints are more accurate than relying on a single camera view alone.

### 3.4. Product Engagement Pipeline

**Figure 6:**
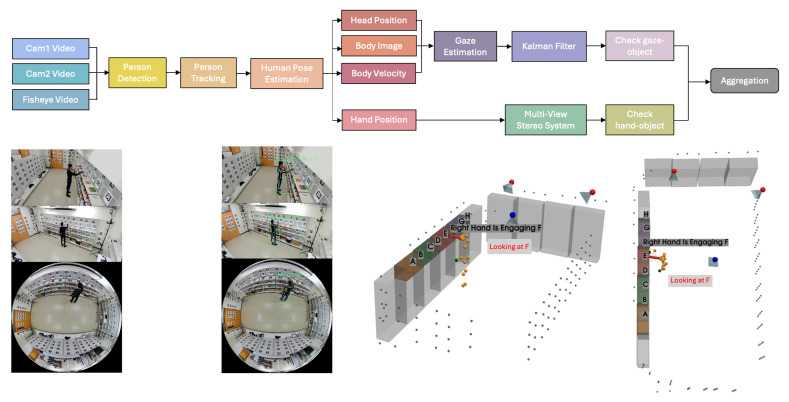
Overview of the product engagement pipeline. The figure illustrates the process of capturing synchronized multi-view video streams to detect and track individuals, estimate human poses and gaze, and localize hand positions. These integrated steps enable the analysis of both visual attention and physical interactions with products in retail environments.

To capture the product engagement using gazes and hands as cues, we propose a product engagement pipeline as illustrated in Figure 6. Our product engagement detection system, including the proposed Transformer-based skeleton encoder for gaze estimation in Section 3.4.4, requires only a fisheye camera and two perspective cameras during deployment. The additional sensors (Pupil Core eye trackers, GoPro cameras, and AprilTag markers) are utilized solely to collect training and evaluation datasets.

The product engagement pipeline begins by capturing synchronized video streams from multiple viewpoints, including side-view cameras (Cam1 and Cam2) and a fisheye camera. These varied perspectives ensure comprehensive coverage of the scene, allowing the system to track individuals and their interactions with products in a retail or similar environment.

The first step in the pipeline involves person detection, which locates every individual in each frame. For perspective-view cameras, we use YOLOX [44] to detect people, while for the fisheye top-view camera frames, we employ RAPID [45], which is specifically designed for human detection in fisheye imagery. Next, person tracking links these detections across consecutive frames, ensuring that each individual can be followed over time, even if they move around the space. To accomplish this, we rely on HybridSort [46], which incorporates an appearance descriptor called CLIP ReID [47] to maintain robust identity tracking despite changes in viewpoint or partial occlusion.

Once each person is tracked, human pose estimation is performed to extract keypoints representing the position of body joints. We use the off-the-shelf human pose estimation model called PCT [48], and from these pose keypoints, we derive additional attributes such as body velocity and head position. These features help capture a person’s movement patterns and orientation over time. They also serve as key inputs for the gaze estimation module, D3DGaze [49], which is adopted from a previous study. Because the predicted 3D gaze from each camera can be uncertain, we integrate the 3D gaze estimates from the three cameras using a Kalman filter [50]. The Kalman filter is particularly useful when variables of interest are only measured indirectly and measurements are available from multiple sensors that may be subject to noise. By providing a probabilistic density function, it enables more reliable estimation and confidence assessment of the continuous 3D gaze trajectories.

In parallel, we localize hand positions using a multi-view stereo approach, combining observations from multiple cameras to track the hands in three-dimensional space. The pipeline checks for hand-object interactions by comparing the hand coordinates with product or shelf positions. This step is critical for detecting physical engagement, such as picking up or returning items.

By capturing where individuals look and how they physically interact with products, the pipeline could provide a measure of which items draw attention and interest during video analysis.

#### 3.4.1. Hand-Object Engagement Pipeline

The estimated 2D keypoints that are obtained from the human pose estimation in three camera views are triangulated to reconstruct the 3D keypoints using the stereo camera technique described in Section 3.3.1. Once their 3D positions are computed, we calculate the overlap between the keypoints of both hands and the shelves. The shelves are voxelized into cubes, and the keypoints of the hands are modeled as spheres; when the fraction of overlapping volume exceeds 0.2, we label it as an interaction. This 0.2 threshold was chosen as a minimal contact criterion based on visual inspection of our annotations; it reliably distinguishes true hand–shelf contacts without being overly sensitive to minor pose variations. Additionally, because conventional skeletal structures localize the hand keypoint at the wrist, we compensate for this distance gap by extending the shelf’s dimension toward the subject by 15 cm. The 15 cm value corresponds to the approximate average distance between the human wrist joint and the midpoint of the middle finger during a reach; this anatomically grounded offset is expected to be robust to small deviations.

#### 3.4.2. Gaze-Object Engagement Pipeline

**Figure 7:**
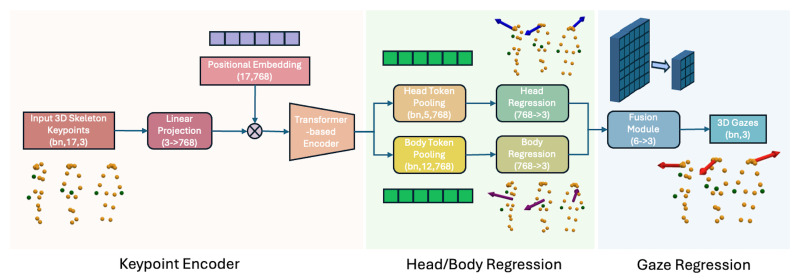
Overview of the Transformer-based skeleton encoder for 3D gaze estimation. This model processes raw 3D skeleton keypoints by first projecting them through a linear layer with positional embeddings to form tokens that are input into a Transformer encoder. The encoder captures spatial relationships among the keypoints to generate a comprehensive skeletal representation. Dedicated regression streams then separately extract head and body features, which are fused to produce the final 3D gaze predictions. The Transformer’s hidden-layer dimension is set to 768 to match the embedding size of CLIP ViT-B-32’s projection head [51], which ensures consistency with established Transformer-based encoder designs. By relying on structural pose information rather than raw RGB data, this approach remains robust against background and environmental variations.

The gaze estimation module, D3DGaze [49], outputs 3D gaze vectors in each camera’s coordinates. These 3D gaze vectors are then transformed into the world coordinates. We combine these estimations together using a Kalman filter. In addition, we experiment with directly estimating 3D gaze vectors from head keypoints as a baseline performance. Image-based gaze estimation can be affected by variations in background and environmental conditions. To address this, we also propose a Transformer-based skeleton encoder for the gaze estimation task. Inspired by the architecture of D3DGaze [49], we design the network architecture as shown in Figure 7. The details of this model are described in Section 3.4.4.

#### 3.4.3. Kalman Filter

The Kalman filter fuses the current noisy measurement of gaze direction $z_{k}$ with the previous state estimate to reduce the overall noise, resulting in a smoother gaze direction estimate.

To transform camera measurements into the world coordinate system, note that each camera produces a 3D gaze vector in its own coordinate system:

(6) $$V_{cam}=\left(\begin{array}{c} v_{x}\\ v_{y}\\ v_{z} \end{array}\right).$$

Given the extrinsic parameters for each camera (with a rotation matrix **R** and translation vector **t**), note that because these gaze vectors are directions (unit vectors) rather than points, the translation is not applied. Instead, the transformation is simply as follows:

(7) $$V_{world}=R^{T}V_{cam}.$$

Here, **R** is the rotation from the world to the camera coordinate. For three cameras (say, fisheye, Cam1, and Cam2), the corresponding transformed measurements are as follows:

(8) $$\begin{array}{cc} V_{world}^{\left(f\right)} & =R_{f}^{T}V_{cam}^{\left(f\right)},\\ V_{world}^{\left(1\right)} & =R_{1}^{T}V_{cam}^{\left(1\right)},\\ V_{world}^{\left(2\right)} & =R_{2}^{T}V_{cam}^{\left(2\right)}, \end{array}$$

A combined measurement $z_{k}$ for frame *k* can be computed by averaging the following:

(9) $$z_{k}=\frac{1}{3}\left(V_{world}^{\left(f\right)}+V_{world}^{\left(1\right)}+V_{world}^{\left(2\right)}\right).$$

For the Kalman filter formulation, we define the state at time *k* as the true gaze direction (in world coordinates):

(10) $$X_{k}=\left(\begin{array}{c} g_{x}\\ g_{y}\\ g_{z} \end{array}\right).$$

Assuming a constant (or slowly varying) gaze direction, we model the system dynamics as a random walk, where each new state is derived from the previous state plus a small, random change:

(11) $$X_{k+1}=FX_{k}+W_{k},$$

where $F=I_{3\times 3}$, and $W_{k}$ is the zero-mean process noise with covariance **Q**. A smaller **Q** implies that the gaze remains nearly constant, while a larger **Q** allows for more rapid changes. The measurement model is as follows:

(12) $$z_{k}=HX_{k}+\eta_{k},$$

where $H=I_{3\times 3}$, and the term $\eta_{k}$ represents measurement noise, assumed to be zero-mean Gaussian:

(13) $$\eta_{k}∼N\left(0,\sigma^{2}\right),$$

with $\sigma^{2}$ is the measurement noise covariance. The Kalman filter consists of two main steps—prediction and update.

- Prediction step:
  (1). State prediction:
    (14) $$\begin{array}{cc} {\hat{X}}_{k|k-1} & =F\;{\hat{X}}_{k-1|k-1} \end{array}$$
  (2). Covariance prediction:
    (15) $$\begin{array}{cc} P_{k|k-1} & =F\;P_{k-1|k-1}\;F^{T}+Q \end{array}$$

- Update step:
  (1). Kalman gain:
    (16) $$\begin{array}{cc} K_{k} & =P_{k|k-1}\;H^{T}{\left(H\;P_{k|k-1}\;H^{T}+\sigma^{2}\right)}^{-1} \end{array}$$
  (2). State update:
    (17) $$\begin{array}{cc} {\hat{X}}_{k|k} & ={\hat{X}}_{k|k-1}+K_{k}\left(z_{k}-H\;{\hat{x}}_{k|k-1}\right) \end{array}$$
  (3). Covariance update:
    (18) $$\begin{array}{cc} P_{k|k} & =\left(I-K_{k}H\right)\;P_{k|k-1} \end{array}$$

- Normalization:

Since $X_{k}$ represents a gaze direction (a unit vector), we normalize the update state:

(19) $$\begin{array}{cc} {\hat{X}}_{k|k}\; & \leftarrow \frac{{\hat{X}}_{k|k}}{∥{\hat{X}}_{k|k}∥}. \end{array}$$

This complete formulation allows us to fuse measurements from multiple cameras, compensating for noise and ensuring a robust and smooth estimation of the 3D gaze direction in world coordinates.

#### 3.4.4. Our Proposed Transformer-Based Skeleton Encoder for Gaze Estimation

This model processes 3D skeleton keypoints to predict a person’s 3D gaze by combining a Transformer-based encoder with separate head and body regression streams as portrayed in Figure 7. First, raw 3D keypoints are projected through a linear layer and enriched with positional embeddings, forming tokens that feed into a Transformer encoder. The encoder captures spatial relationships among the keypoints and generates a rich representation of the entire skeleton. Because the approach relies on human pose estimation skeletons rather than raw RGB inputs, it is largely invariant to background changes and other visual variations. From the encoded tokens, the model pools specific features for the head and body, separately regressing each to capture finer details of head orientation and body pose. Finally, a fusion module combines the head and body regressions into a single representation, which is used to produce the final 3D gaze predictions. This skeletal-based method is advantageous over purely RGB-based models because it focuses on structural information of the body rather than appearance, thereby improving robustness to lighting, clutter, and other environmental factors.

### 3.5. Dataset for Fine-Tuning Gaze Estimator and Our Proposed Model

**Figure 8:**
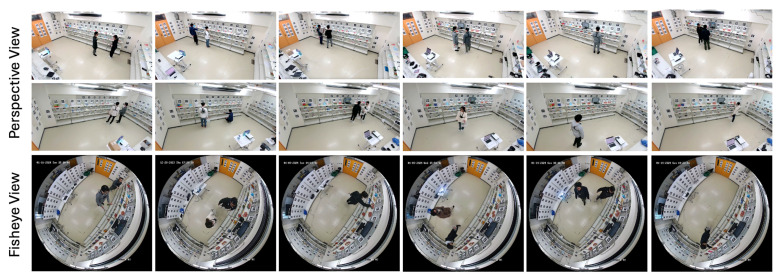
Examples from the fine-tuning dataset. The figure displays sample frames captured by the fisheye and perspective cameras in our simulated retail setup, featuring participants wearing eye trackers and chest-mounted GoPro cameras.

Since the adopted gaze estimation model was trained on the GAFA dataset [49], which features different environments and camera views than our simulated retail setup, we collected a new dataset using a fisheye camera and two perspective cameras. The additional sensors, such as the eye trackers and GoPro cameras, were used solely to generate ground truth labels and supplementary data for training and fine-tuning the gaze estimation models. For actual deployment and estimation, only the fisheye camera and the two perspective cameras are required. Thirteen participants wore eye trackers and GoPro cameras on their chests, and each recording features one or two people in the scene. Figure 8 shows examples from the dataset, and Table 2 provides detailed information. The fine-tuning dataset was randomly split at the video level into training, validation, and test sets. Each video was assigned to one set to prevent frame-level data leakage.

Fine-tuning in this paper refers to the process of adapting pretrained models, both the D3DGaze gaze estimation model and our proposed Transformer-based skeleton encoder, to our specific retail environment. By re-training these models on our newly collected dataset, which includes fisheye and perspective camera views, reconstructed 3D skeleton keypoints, and ground truth head, body, and gaze directions, fine-tuning allows the models to adjust their parameters to better capture the unique characteristics and challenges of our simulated retail setup. This targeted adaptation could help enhance model accuracy and robustness for detecting and analyzing product engagement.

**Figure 9:**
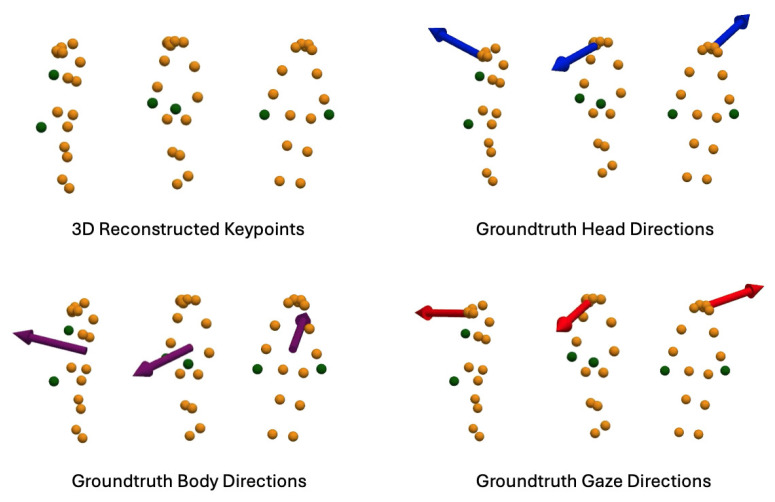
Examples from the fine-tuning dataset for the Transformer-based skeleton encoder. This dataset comprises reconstructed 3D skeleton keypoints, ground truth head directions from the eye tracker’s world camera, ground truth body directions from the GoPro camera, and ground truth gaze directions from the eye tracker’s eye cameras.

Using the same dataset, we reconstruct 3D skeleton keypoints from the estimated 2D keypoints to fine-tune our Transformer-based skeleton encoder for gaze estimation. This dataset includes 3D skeleton keypoints, ground truth head directions from the eye tracker’s world camera, ground truth body directions from the GoPro camera, and ground truth gaze directions from the eye tracker’s eye cameras. Examples are shown in Figure 9.

The ground truth 3D gaze direction is derived from the Pupil Core eye tracker’s eye camera data, calibrated using the standard pupil-to-world mapping provided by the manufacturer. Each gaze vector is computed by intersecting the 3D line of sight from the pupil center with the scene captured by the world camera, and then transformed into the world coordinate system using known extrinsic parameters. In our setup, we use the physical calibration marker provided by Pupil Labs to perform spatial calibration. This marker enables precise mapping between the pupil position in the eye camera and the 3D environment seen by the world camera. The resulting transformation is used to register all gaze vectors into the global coordinate system. According to the Pupil Labs documentation, the 3D gaze estimation error of the pupil core system is typically around 1.5–2.5 degrees under optimal conditions. In our setup, we assume a similar level of accuracy. To ensure reliable ground truth data, we excluded frames with confidence scores below 0.6, the built-in default threshold from the Pupil Core SDK for filtering out samples affected by occlusions, blinks, or calibration instability.

### 3.6. Product Engagement Dataset

**Figure 10:**
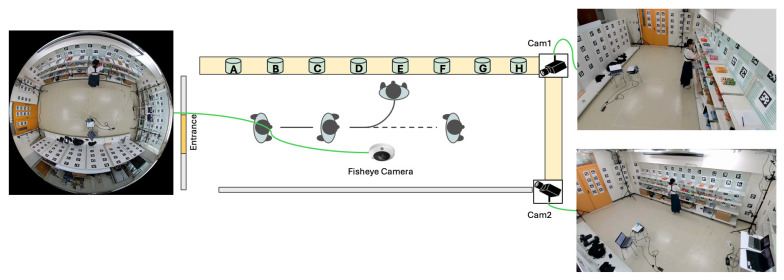
Overview of the product engagement data collection setup. This figure displays the arrangement of eight product item categories (A–H) on the top of shelves within the experimental environment.

To evaluate our proposed product engagement system and the Transformer-based skeleton encoder for gaze estimation, we collected data in five different scenarios in which a subject engages with objects by either looking at them or picking them from the shelves. In this experiment, eight different item categories (product items A through H) are placed on the top of the shelves, as shown in Figure 10. This dataset is specifically designed to assess the performance of both the hand-object pipeline (which evaluates physical interactions) and the gaze-object pipeline (which evaluates visual engagement) in detecting product engagement.

**Figure 11:**
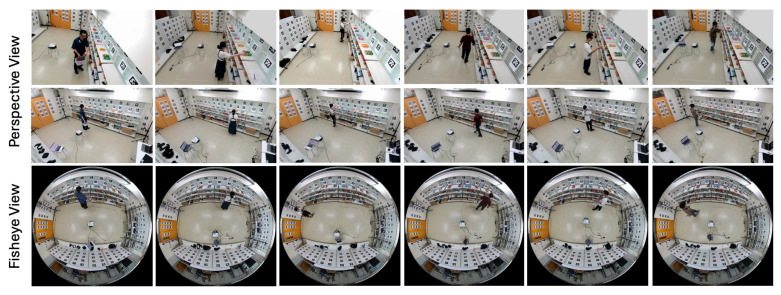
Sample frames from the product engagement dataset captured in both perspective and fisheye views. This figure illustrates subjects interacting with products via gaze and hand actions within the simulated retail environment.

Six participants wore eye trackers and chest-mounted GoPro cameras, with each recording featuring only one participant. These additional sensors were only employed to provide ground truth labels for evaluation purposes. In each scenario, the subject first checks out the items on the gaze-object list and then proceeds to pick the items on the hand-object list (see Table 3). There were no predefined rules regarding gaze behavior; participants were simply instructed to search for and pick up specified items, and were allowed to move and look around naturally. This design aimed to preserve realistic and spontaneous engagement behavior. Detailed information about this dataset is provided in Table 4, and sample frames are presented in Figure 11.

**Figure 12:**
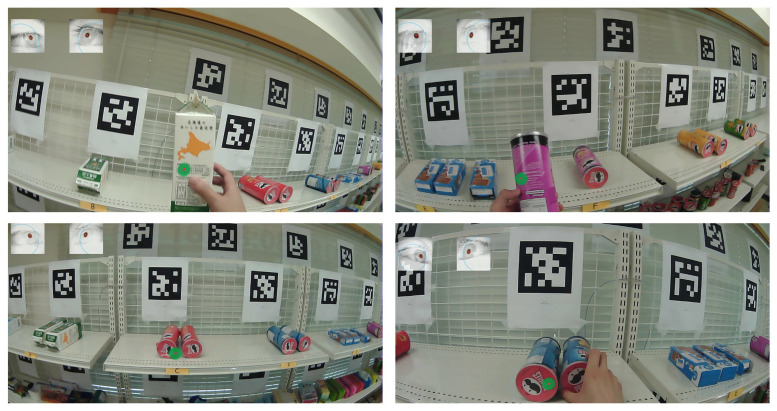
Visualization of gaze points collected by the eye trackers (localized in world coordinates using AprilTag markers), overlaid on the scene. This figure demonstrates how gaze annotations correspond to product regions on the shelves and validates cases where the subject continues to gaze at a product after picking it up.

For frame-wise annotation of both hand-object and gaze-object engagement, we inspect each frame individually and label the engaged product based on specific criteria. For hand-object engagement, a product is annotated as engaged when (1) the subject’s hand is within the shelf area where the product is located, and (2) the subject holds the product after picking it from the shelf. For gaze-object engagement, a product is annotated as engaged if either (1) the subject’s gaze falls on the product within its designated shelf region, or (2) the gaze point is directly located on a product that the subject is holding. To quantify this, we compute the overlapping volume between the ground truth gaze vectors and the voxelized regions corresponding to the shelves where the products are placed. We further validate these frame-wise annotations by comparing them with the gaze points collected by the eye trackers, as illustrated in Figure 12, which also highlights instances where the subject continues to gaze at a product after picking it up.

## 4. Results

In this section, we present our quantitative and qualitative evaluations. We first assess the accuracy of 3D keypoint reconstruction, then compare gaze estimation methods, and finally evaluate the hand-object and gaze-object engagement detection pipelines within our proposed product engagement detection system. We conclude with an analysis of how the aggregation of hand and gaze signals provides richer insights into customer behavior.

### 4.1. Evaluation for 3D Reconstruction of Keypoints

To assess the accuracy of our multi-camera 3D reconstruction, we compare the ground truth 3D keypoints $x_{i}$ with the estimated positions ${\hat{x}}_{i}$ obtained from various camera configurations in the dataset described in Section 3.3. Two main error metrics are used, namely, the mean squared error (MSE) and the root mean squared error (RMSE). As defined in Equations (20) and (21), MSE measures the average of the squared distance between ground truth and reconstructed keypoints, whereas RMSE, being the square root of MSE, provides an error value in the same units as the original 3D data (millimeters).

(20) $$\mathrm{MSE}=\frac{1}{N}\sum_{i=1}^{N}{∥x_{i}-{\hat{x}}_{i}∥}^{2},$$

(21) $$\mathrm{RMSE}=\sqrt{\mathrm{MSE}},$$

From Table 5, ‘All Cameras’ refers to incorporating all available camera view results in an MSE of 0.000524 m^2^ and an RMSE of 22.89 mm. This relatively low error indicates that the reconstruction algorithm benefits from a richer set of viewpoints. The additional perspectives help reduce ambiguities in depth and occlusion, leading to more precise triangulation and, consequently, higher accuracy in the 3D keypoint estimates.

Cam1 and Fisheye: When only Cam1 and the fisheye camera are used, the MSE is slightly higher at $0.000576\;m^{2}$ (RMSE of 24.01mm). Although still reasonably accurate, losing one perspective camera view can increase uncertainty in certain regions, especially where occlusions might occur or where the fisheye camera has limited resolution in peripheral areas.

Cam2 and fisheye: This combination exhibits a more pronounced increase in error, with an MSE of $0.003501\;m^{2}$ (RMSE of 59.17mm). The difference suggests that Cam2’s field of view and the fisheye camera’s coverage may not overlap as effectively for precise triangulation, or that the geometry between these two viewpoints is less optimal for certain keypoints, resulting in a higher overall reconstruction error.

Cam1 and Cam2: Interestingly, this pairing achieves an exceptionally small MSE of $9.90\times 10^{-17}\;m^{2}$ and an RMSE of $9.95\times 10^{-6}\;\mathrm{mm}$. Such near-zero errors may indicate near-perfect alignment in a subset of data, an ideal camera placement for the observed scene, or potential measurement artifacts (e.g., simplified ground truth conditions). Further investigation of this scenario would clarify whether it arises from near-ideal camera geometry or particular data collection conditions.

Overall, these results demonstrate that more diverse or complementary camera views generally lead to more accurate 3D keypoint reconstruction. As both our hand-object detection pipeline and the proposed Transformer-based skeleton encoder for gaze estimation rely entirely on these reconstructed 3D keypoints (triangulated from estimated 2D keypoints), any error in reconstruction propagates directly into downstream engagement detection. The MSE and RMSE metrics clearly show how reducing the number or variety of camera angles can introduce uncertainty, especially in scenes where occlusions or depth ambiguities are common. Notably, the consistently low MSE and RMSE values are largely a result of the robust multi-camera calibration technique developed in our previous study [43]. By carefully selecting camera placements or integrating additional viewpoints, we can significantly improve the reliability and precision of 3D human keypoint estimation.

### 4.2. Evaluation of Gaze Estimation on the Fine-Tuning Dataset

To evaluate the performance of our proposed skeleton-to-gaze model under controlled conditions, we first assess its accuracy on the test split of the fine-tuning dataset introduced in Section 3.5. The model achieves a 3D mean angular error (MAE) of 29.83° and processes frames at a speed of 2401.32 FPS on a high-performance system equipped with an Intel Core i9-10940X CPU and a single NVIDIA RTX A6000 GPU (48 GB VRAM).

To further investigate the contribution of individual loss terms, we conducted an ablation study by evaluating two modified variants of our model, that is, one with the head loss removed and another with the body loss removed. The full loss function used in training consists of three components—head loss, body loss, and gaze loss—following the formulation in [49] (Table 6).

The ablation results show that both head and body loss components contribute meaningfully to the final model performance. Removing either component results in reduced accuracy, with body loss having a more significant negative impact. Although our method demonstrates notably faster inference than D3DGaze, it is important to emphasize that these results were obtained on high-end hardware and do not directly imply suitability for deployment in real-time or cost-sensitive environments.

It is also worth noting that some scenes in the test set were recorded using only two perspective cameras. In such cases, 3D skeletons were reconstructed from 2D keypoints estimated from the available views. For D3DGaze, which operates on per-camera image inputs, 3D gaze vectors were first estimated in each view, transformed into the global coordinate system, and then fused using a Kalman filter as described in Section 3.4.3.

### 4.3. Evaluation of Gaze Estimation on the Product Engagement Dataset

We evaluate several gaze estimation methods on our product engagement dataset using the 3D mean angular error (MAE) metric. This evaluation is critical for our gaze-object detection pipeline because accurate gaze estimation underpins the entire detection process; small deviations in the estimated gaze can lead to incorrect assessments of which products are capturing a customer’s visual attention. Ensuring high precision in gaze estimation directly contributes to the robustness and reliability of detecting gaze-object engagement. MAE is computed as the average angular difference (in degrees) between the ground truth and predicted 3D gaze vectors:

(22) $$\mathrm{MAE}=\frac{1}{N}\sum_{i=1}^{N}\theta_{i},$$

where $\theta_{i}$ is the angular difference (in degrees) between the ground truth gaze vector and the predicted gaze vector for the *i*-th sample.

Table 7 summarizes the mean angular errors of several gaze estimation approaches evaluated on the product engagement dataset from Section 3.6. The head keypoints baseline, which relies only on head pose information, achieves a 3D MAE of 35.50°. By contrast, the skeleton-to-gaze method, our proposed Transformer-based skeleton encoder for gaze estimation, significantly reduces the error to 29.13°, benefiting from a more holistic body representation. We also compare two variants of the D3DGaze model, namely, D3DGaze (NF), which is used without fine-tuning, and D3DGaze, which is fine-tuned on our dataset described in Section 3.5. Interestingly, D3DGaze without fine-tuning (23.31°) performs better than the fine-tuned version (25.33°), indicating that the pretrained weights can generalize well to new data. Notably, our Transformer-based skeleton-to-gaze model, pretrained on the same dataset without further fine-tuning, demonstrates competitive performance. Overall, these results highlight the benefits of leveraging full skeletal cues, as well as the importance of large-scale pretraining and proper fine-tuning strategies for accurate 3D gaze estimation.

**Figure 13:**
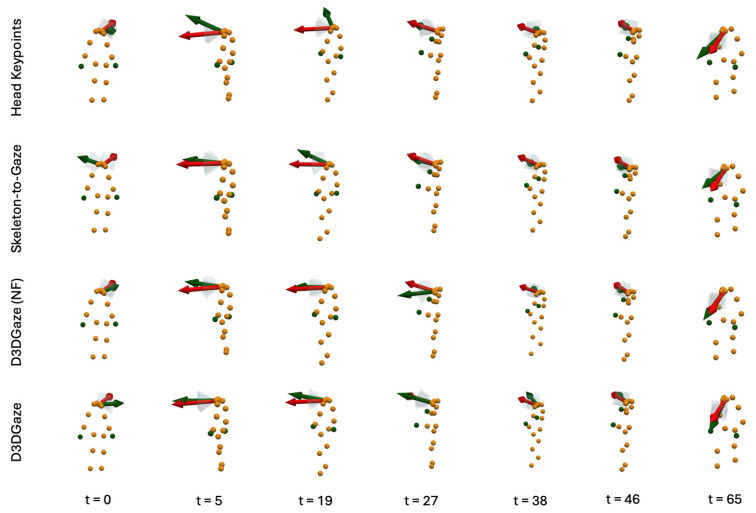
Qualitative comparison of gaze estimation methods. Each column represents a different time frame *t*, while each row corresponds to a specific gaze estimation method. Orange dots indicate the subject’s 3D skeleton keypoints, red arrows denote the ground truth gaze direction, and green arrows represent the model’s predicted gaze direction.

In the visual comparisons portrayed in Figure 13, each column corresponds to a different time frame t, while each row represents a particular gaze estimation method. The orange dots indicate the 3D skeleton keypoints of the subject, the red arrows represent the ground truth gaze direction, and the green arrows depict the model’s predicted gaze direction.

The head keypoints (first row) baseline relies primarily on the head pose to infer the gaze. As seen in several frames, the predicted gaze (green) often deviates considerably from the ground truth gaze (red), especially when the head orientation alone is insufficient to capture subtle changes in eye direction. The skeleton-to-gaze method (second row) leverages the entire body pose rather than only the head region. By using more comprehensive skeletal cues, the method typically yields predictions that align more closely with the red arrows. Nevertheless, occasional discrepancies remain, particularly in challenging poses or rapid head movements, such as at t=0. D3DGaze (NF) (third row) is the D3DGaze model without fine-tuning. Despite the lack of domain-specific adjustments, the green arrows often match the red arrows quite well, indicating that the pretrained model can generalize effectively to new data. D3DGaze (fourth row) is the same model, but with fine-tuning on the dataset described in Section 3.5. While it also demonstrates solid alignment in most frames, some columns show slight divergences from the ground truth. These differences can stem from the updated parameters overfitting certain aspects of the dataset or not fully capturing all the nuances of the gaze variations. Overall, the figure illustrates how each method handles different viewpoints and poses across time. The skeleton-to-gaze method and both D3DGaze variants generally track more consistent gaze directions than the head-only method, highlighting the benefits of richer body context. Meanwhile, the fine-tuning process can either refine or alter a model’s learned representation, as evidenced by the subtle variations between D3DGaze (NF) and D3DGaze.

### 4.4. Evaluation of Hand-Object Detection on the Product Engagement Dataset

To evaluate the hand-object engagement detection, each video frame is annotated with one of the labels {A,B,C,D,E,F,G,H,None}. We use only those frames where product engagement occurs (i.e., frames labeled with a product label) because our goal is to identify when a hand physically interacts with a product, we restrict evaluation to frames annotated with one of the eight product labels, and frames labeled “None” (no engagement) are excluded from metric calculations. A missed detection occurs when the ground truth is a product label (e.g., *A*) but the model predicts “None”, while a false detection happens if the model predicts a product label erroneously. We use three key evaluation metrics: accuracy, balanced accuracy, and weighted F1 score.

Accuracy is defined as the fraction of frames where the predicted label exactly matches the ground truth label:

$$\mathrm{Accuracy}\;=\;\frac{1}{N}\sum_{i=1}^{N}1\left({\hat{y}}_{i}=y_{i}\right),$$

where

- *N* is the total number of frames evaluated.
- $y_{i}$ is the true label for frame *i*.
- ${\hat{y}}_{i}$ is the predicted label for frame *i*.
- 1(·) is the indicator function that equals 1 if its argument is true, and 0 otherwise.

Accuracy gives an overall measure of the model’s correctness. However, in imbalanced scenarios, high accuracy might be achieved even if the model fails to correctly identify an engaged product.

Balanced accuracy addresses class imbalance by averaging the recall across all classes:

$$\mathrm{Balanced}\;\mathrm{accuracy}\;=\;\frac{1}{C}\sum_{c=1}^{C}\frac{{\mathrm{TP}}_{c}}{{\mathrm{TP}}_{c}+{\mathrm{FN}}_{c}},$$

where

- *C* is the total number of classes (here, 8 classes corresponding to A,B,C,D,E,F,G, and *H*).
- ${\mathrm{TP}}_{c}$ is the number of true positives for class *c*.
- ${\mathrm{FN}}_{c}$ is the number of false negatives for class *c*.

Balanced accuracy ensures that each class contributes equally to the final metric, regardless of how frequently it appears.

The weighted F1 score is the weighted average of the F1 scores computed for each class. The F1 score for class *c* is the harmonic mean of its precision and recall:

$${F1}_{c}\;=\;2\times \frac{{\mathrm{Precision}}_{c}\times {\mathrm{Recall}}_{c}}{{\mathrm{Precision}}_{c}+{\mathrm{Recall}}_{c}},$$

with

$${\mathrm{Precision}}_{c}\;=\;\frac{{\mathrm{TP}}_{c}}{{\mathrm{TP}}_{c}+{\mathrm{FP}}_{c}}\;\mathrm{and}\;{\mathrm{Recall}}_{c}\;=\;\frac{{\mathrm{TP}}_{c}}{{\mathrm{TP}}_{c}+{\mathrm{FN}}_{c}}.$$

The weighted F1 score is then defined as follows:

$$\mathrm{Weighted}\;F1\;=\;\sum_{c=1}^{C}w_{c}\;\times \;{F1}_{c},$$

where the weight $w_{c}$ for class *c* is given by the following:

$$w_{c}\;=\;\frac{n_{c}}{N},$$

with $n_{c}$ being the number of frames with the ground truth label *c*. This metric reflects both the balance between precision and recall, and the importance (or prevalence) of each class in the overall dataset.

In summary, accuracy gives a general measure of overall correctness, but it can be misleading in imbalanced datasets. Balanced accuracy ensures that all classes (including rare product labels) are treated equally by averaging per-class recall. The weighted F1 score provides a more nuanced view by combining precision and recall into one metric, while weighting each class by its frequency. These metrics together offer a comprehensive evaluation of our model’s performance in detecting hand-object or gaze-object engagement, particularly highlighting its ability to correctly identify product engagement.

The results represented in Table 8 provide a comprehensive view of how different camera configurations perform on the hand-object engagement detection task for the “label shelf” annotation. The asterisk “*” denotes the configurations where a 15 cm distance compensation is applied to the wrist skeleton and hand location. As described in Section 3.6, in our annotation process for both hand-object and gaze-object engagement detection, frames are annotated as “label shelf” when the subject’s hand or gaze is detected within the shelf area where a product is placed, and as “label product” when the subject is observed holding the product after picking it up; the “label both” annotations are then generated by combining the frames labeled as “label shelf” and “label product”, with frames that do not meet either criterion labeled as “None”. The table summarizes key metrics—accuracy, balanced accuracy, and weighted F1—across 2440 frames for the “label shelf” annotated frames for hand-object engagement.

The All Cameras configuration achieves an accuracy of 79.1% and a balanced accuracy of 81.3%, with a weighted F1 score of 87.2%. This strong performance can be largely attributed to the accurate 3D reconstruction of keypoints, which provides precise spatial information for the hand-object detection pipeline. The All Cameras* version outperforms the All Cameras version with an accuracy of 83.9%, balanced accuracy of 85.5%, and weighted F1 of 89.9%.

In contrast, configurations that exclude specific camera components, such as Cam1, Cam2, FisheyeCam2, and FisheyeCam1, show a clear drop in performance. For instance, the Cam1,Cam2 configuration has an accuracy of only 59.9% and a balanced accuracy of 60.6%, with a weighted F1 of 67.9%. This indicates that the fisheye view is important for accurately reconstructing the 3D skeleton keypoints from estimated 2D keypoints. Similarly, the removal of individual cameras (as seen in FisheyeCam2 and FisheyeCam1) also degrades performance, with FisheyeCam2 performing particularly poorly (accuracy of 58.1%) compared to FisheyeCam1 (accuracy of 70.0%). The wrist-hand distance compensated versions of these configurations (Cam1,Cam2*, FisheyeCam2*, and FisheyeCam1*) show some recovery in performance, yet they still do not reach the level of the All Cameras or All Cameras* setups.

**Figure 14:**
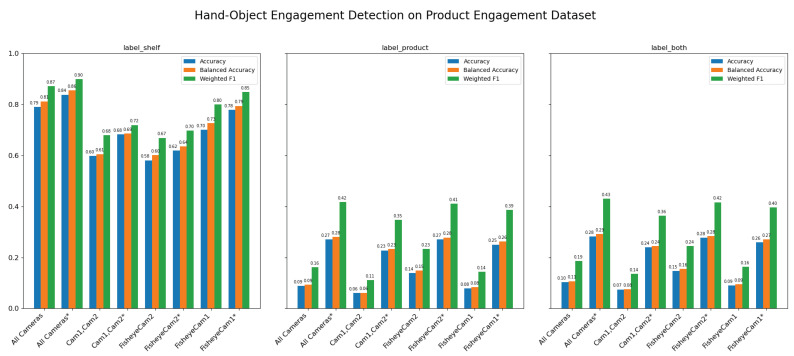
Comparison of accuracy, balanced accuracy, and weighted F1 across three evaluation cases (label shelf, label product, and label both) for each camera configuration. Our method still struggles to detect engagement once a product is held, as reflected by lower scores in the “label” product and “label both” cases. The asterisk “*” denotes the configurations where a 15 cm distance compensation is applied to the wrist skeleton and hand location.

Figure 14 compares three performance metrics, accuracy, balanced accuracy, and weighted F1, across three evaluation cases (label shelf, label product, and label both) for each camera configuration. Each subplot corresponds to a different evaluation case, with the bars in each group representing the three metrics. In general, the wrist-hand distance–compensated versions show higher performance, particularly in terms of accuracy and weighted F1. Conversely, removing certain cameras tends to reduce performance, as seen by the shorter bars for those configurations. Overall, these results suggest our method can accurately detect hand-object engagement when a subject’s hand interacts with a product on the shelf, however, still lacks the ability to detect hand-object engagement after the product has been picked from the shelf and held by the subject, as indicated by the drop in performance in the “label product” and “label both” cases.

**Figure 15:**
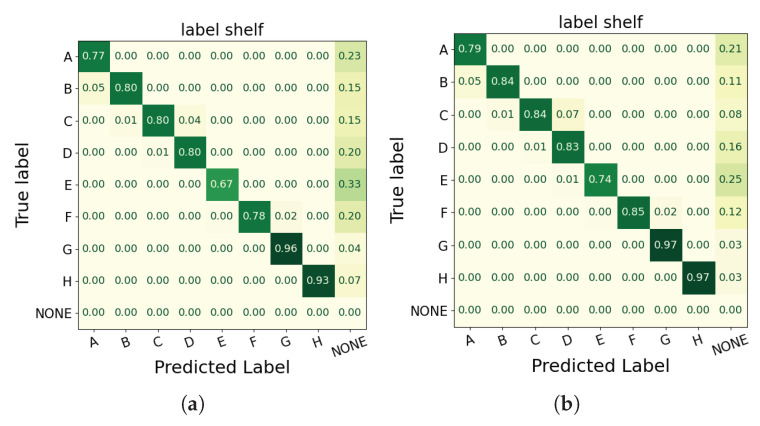
Normalized confusion matrices for hand-object engagement (label shelf case) using a green color scale: darker greens indicate higher values, while lighter greens indicate lower values. (**a**) All Cameras configuration. (**b**) All Cameras* configuration. The higher diagonal values in (**b**) indicate improved classification accuracy and fewer misclassifications for product labels; however, these matrices do not capture the false-positive detections observed in Figure 16, as they only evaluate frames with a product ground truth annotation.

**Figure 16:**
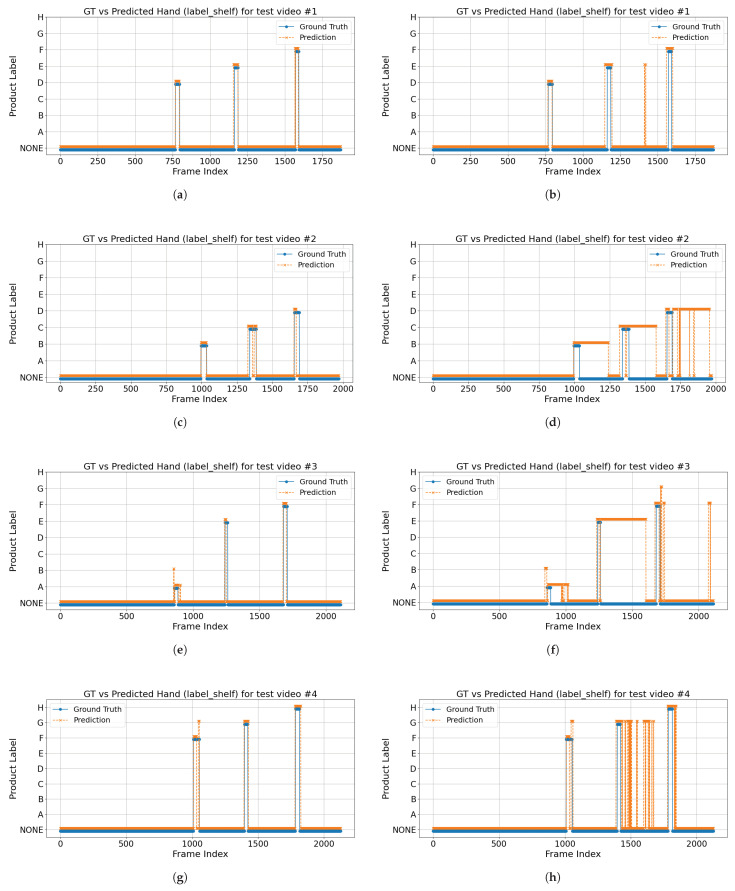
Comparison of detected hand-object engagement versus ground truth for the label shelf case across four test videos. The orange dashed line indicates the predicted shelf labels, while the blue solid line represents the ground truth over consecutive frames. Plots (**a**,**c**,**e**,**g**) correspond to the All Cameras configuration, and plots (**b**,**d**,**f**,**h**) correspond to the All Cameras* configuration.

The two figures in Figure 15 are normalized confusion matrices for the hand-object engagement in the label shelf case, where rows represent the ground truth labels and columns show the predicted labels. Darker diagonal cells indicate more accurate classifications, while lighter off-diagonal cells reveal errors. The first matrix in Figure 15a corresponds to the All Cameras version, and the second matrix in Figure 15b to the All Cameras* version. Comparing the two shows that the All Cameras* configuration produces higher diagonal values for each product label (e.g., A, B, F, H) and fewer “NONE” predictions, signifying fewer misclassifications overall. However, as portrayed in Figure 16, the All Cameras* configuration exhibits significant false-positive detections. These discrepancies are not reflected in the confusion matrices, which only evaluate frames with a product ground truth annotation.

The eight plots in Figure 16 illustrate, for four test videos recorded with the All Cameras and All Cameras* configurations, how the detected hand-object engagement in the label shelf case (orange dashed line) aligns with the ground truth (blue solid line) over consecutive frames. The horizontal axis represents the frame index, while the vertical axis indicates the detected shelf label (A–H) or “NONE”. Most frames remain at “NONE” (no engagement); when a subject’s hand engages a product, the ground truth jumps from “NONE” to a specific label (e.g., “A”, “F”, or “H”), and ideally, the predicted line follows this pattern. The plots show that the All Cameras configuration largely matches the ground truth, whereas the All Cameras* configuration exhibits significant false-positive detections (i.e., it predicts a product label when the ground truth is “NONE”) due to the 15 cm distance compensation applied to the wrist keypoint and hand location. These discrepancies are not reflected in Table 8 and the confusion matrices in Figure 15, which only evaluate frames with a product ground truth annotation.

While distance compensation can reduce missed detections at the precise moment of shelf contact, it also pushes predicted hand positions into the shelf region prematurely, resulting in substantially more false alarms. Consequently, the uncompensated All Cameras configuration remains superior for reliably detecting hand-object engagement across complete video sequences.

We evaluated the impact of applying the 15 cm distance compensation (“All Cameras*”) versus the original multi-camera configuration (“All Cameras”) across all videos in the product engagement dataset, treating hand-object engagement as a binary detection task (engaged vs. none). Binarizing simplifies our analysis to focus purely on whether the model falsely predicts engagement (false positives) or misses it (false negatives), without conflating errors in identifying specific product labels. Across all videos, All Cameras* produced a significantly higher mean frame-level false-positive rate (0.157±0.108) compared to All Cameras (0.092±0.093; paired *t*-test *t* = –2.65, *p* = 0.014). Correspondingly, All Cameras* achieved a lower mean binary F1 score (0.558±0.192) versus All Cameras (0.640±0.196; *t* = 2.71, *p* = 0.012). These statistically significant differences demonstrate that, although distance compensation was intended to reduce missed detections, it instead substantially increases spurious engagement predictions and degrades overall detection performance, confirming that the uncompensated All Cameras configuration is the superior choice for reliable hand-object engagement detection.

**Figure 17:**
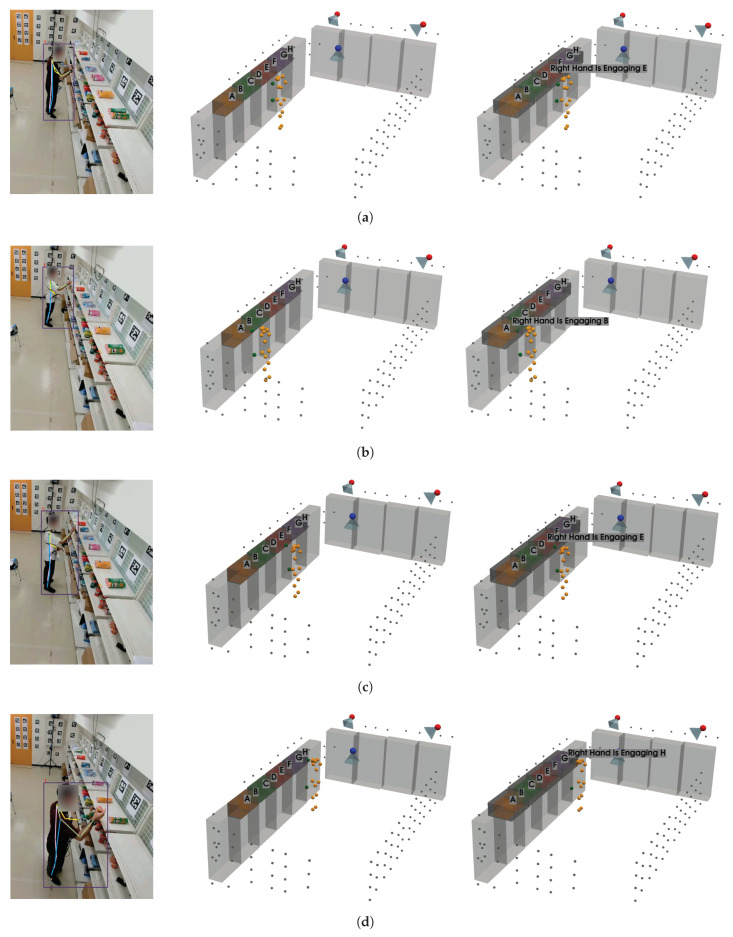
Example frames from test videos illustrating false-positive hand-object engagement detections; (**a**) is from test video 1, frame 1400, where the false-positive detection occurs (“E”); (**b**) is from test video 2, frame 1080, where the false-positive detection occurs (“B”); (**c**) is from test video 3, frame 1300, where the false-positive detection occurs (“E”); and (**d**) is from test video 4, frame 1830, where the false-positive detection occurs (“H”).

To investigate false-positive detections, Figure 17 presents example frames from the test videos where these detections occur, as indicated in Figure 16. The image on the left is from Cam1, the middle image shows the reconstructed 3D keypoints using the All Cameras configuration, and the image on the right shows the reconstructed 3D keypoints using the All Cameras* configuration. It can be observed that these false-positive hand-object engagement detections are caused by distance compensation, as subjects hold product items and check them in front of the shelves from which they were picked. Consequently, these scenarios result in false-positive hand-object engagement detections when distance compensation is applied.

### 4.5. Evaluation of Gaze-Object Detection on the Product Engagement Dataset

Similarly, since our goal is to measure only true instances of visual engagement, we restrict evaluation to frames annotated with a product label and exclude all “None” frames from the metric calculations. Our ability to achieve gaze-object engagement detection is enabled by the adopted gaze estimation pipeline, D3DGaze (with Kalman filtering), and our proposed transformer-based skeleton-to-gaze encoder built on robust reconstructed 3D skeleton keypoints. Table 9 presents the results of gaze-object engagement detection, comparing different methods across three evaluation scenarios: (1) frames in which the subject’s gaze is directed at products on the shelf, (2) frames in which the subject holds the product, and (3) a combined set encompassing both cases. It is important to note that gaze-object engagement detection is computed by measuring the overlapped volume between the gaze vectors and the shelf’s voxel volumes. Consequently, when the product is not on the shelf, such as when the subject is holding it, the overlap is reduced, leading to lower detection metrics.

The head keypoints baseline, which relies solely on head pose information, consistently exhibits lower accuracy and F1 scores, indicating that it struggles to capture the nuances of gaze engagement. In contrast, D3DGaze (NF), the D3DGaze model used without fine-tuning, demonstrates considerable improvements, showcasing the generalization ability of a pretrained gaze estimation model. Fine-tuning the same model (D3DGaze) further enhances performance, particularly in the label shelf and label product frames. This improvement occurs because subjects tended to hold products and check them in front of the shelves where they selected them.

Additionally, the skeleton-to-gaze method, which leverages full skeletal cues via a Transformer-based skeleton encoder, achieves competitive results across all evaluation scenarios. Even though the evaluation of the gaze estimation task suggests that the overall mean angular errors of D3DGaze with fine-tuning and the skeleton-to-gaze are higher. This is likely due to frames in which subjects are not engaging with the shelves or are standing far from them.

**Figure 18:**
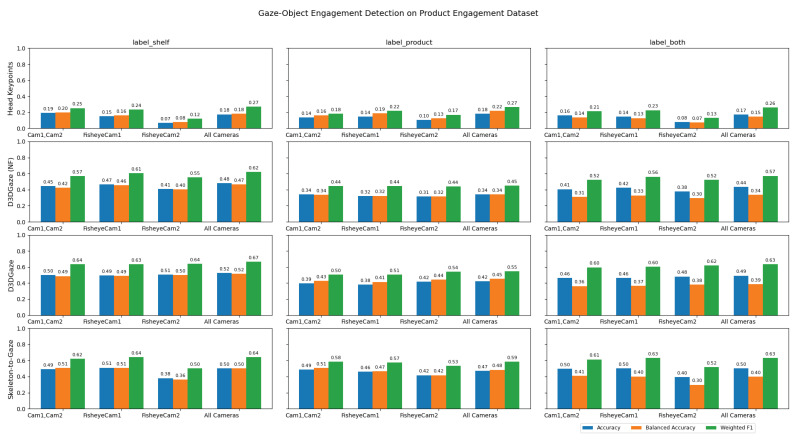
Performance of gaze-object engagement detection across various camera configurations and labeling scenarios, evaluated by accuracy (blue), balanced accuracy (orange), and weighted F1 (green).

Figure 18 illustrates how different camera configurations and labeling scenarios (label shelf, label product, label both) affect the performance of gaze-object engagement detection, measured by accuracy (blue), balanced accuracy (orange), and weighted F1 (green). Along the horizontal axis, configurations such as “Cam1,Cam2”, “FisheyeCam1”, “FisheyeCam2”, and “All Cameras” compare how many and which camera views are used. In general, the label shelf case yields higher scores than the label product case because the method computes engagement based on the overlap between gaze vectors and the shelf’s voxel volumes; if the product is not on the shelf (i.e., the subject is holding it), which overlap naturally decreases. The combined scenario (label both) typically shows intermediate performance, reflecting the mixture of label shelf and label product frames. Moreover, using additional cameras (e.g., “All Cameras”) consistently improves the metrics, emphasizing the importance of multiple viewpoints for reducing ambiguity in gaze estimation. Overall, the figure highlights that the camera configuration choice plays a critical role in determining the system’s accuracy, balanced accuracy, and weighted F1 for gaze-object engagement detection.

**Figure 19:**
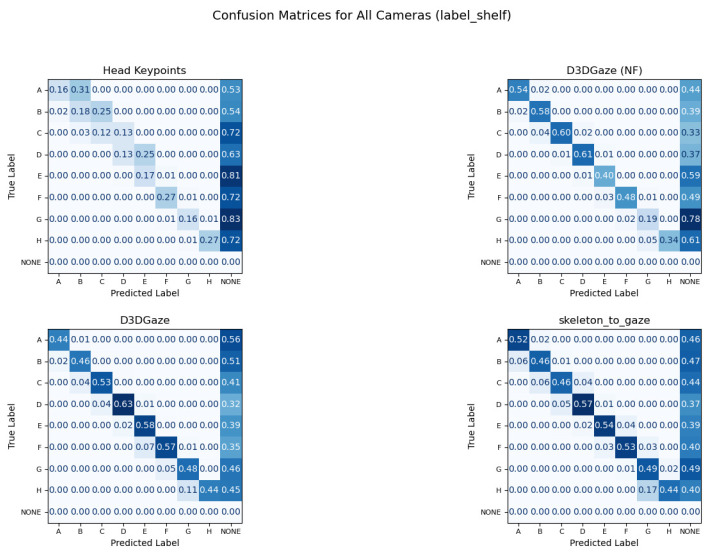
Confusion matrices for gaze-object engagement classification using a blue color scale: darker blues indicate higher values, while lighter blues indicate lower values. Confusion matrices for four gaze estimation methods reveal challenges in accurately classifying gaze-object engagement, with frequent misclassifications evident from low diagonal values.

In Figure 19, the confusion matrices illustrate that each method—head keypoints, D3DGaze (NF), D3DGaze, and skeleton-to-gaze—faces challenges in accurately classifying shelf engagement across various labels. In several cases, the diagonal values remain below 0.50, indicating frequent misclassifications. This is particularly evident when the estimated gaze directions fail to overlap with the shelves’ voxels, likely due to errors in the gaze estimation methods. Overall, the performance across these four approaches shows room for improvement. For example, the head keypoints method struggles to differentiate closely related classes, and the D3DGaze methods, despite leveraging 3D gaze information, still exhibit significant errors that result in poor overlap with the shelves’ voxels. Even skeleton-to-gaze, which incorporates body pose data, demonstrates only moderate success in aligning the estimated 3D gaze directions with the shelves’ voxels. These results suggest that further refinement, such as improved feature extraction, more robust training data, or better model architectures, is needed to reliably detect gaze-object engagement in real-world scenarios.

**Figure 20:**
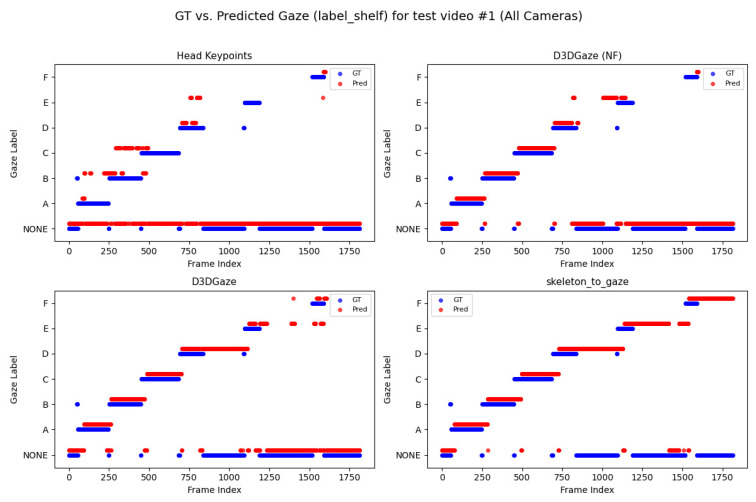
Comparison of ground truth (red) and predicted (blue) gaze directions for the label shelf case on test video 1.

**Figure 21:**
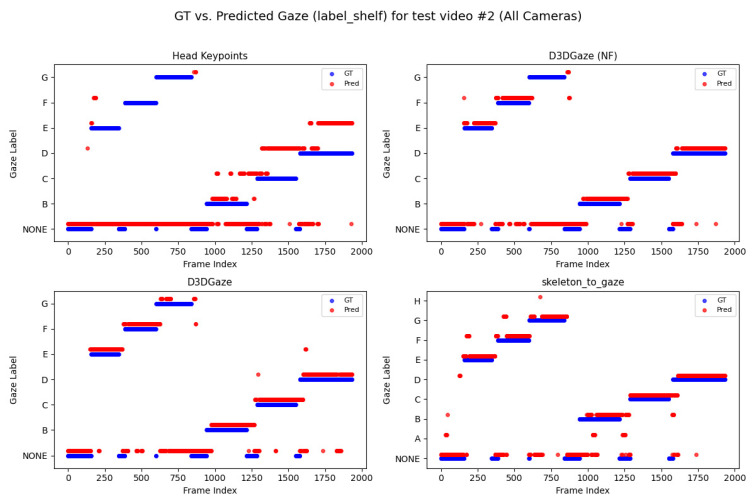
Comparison of ground truth (red) and predicted (blue) gaze directions for the label shelf case on test video 2.

**Figure 22:**
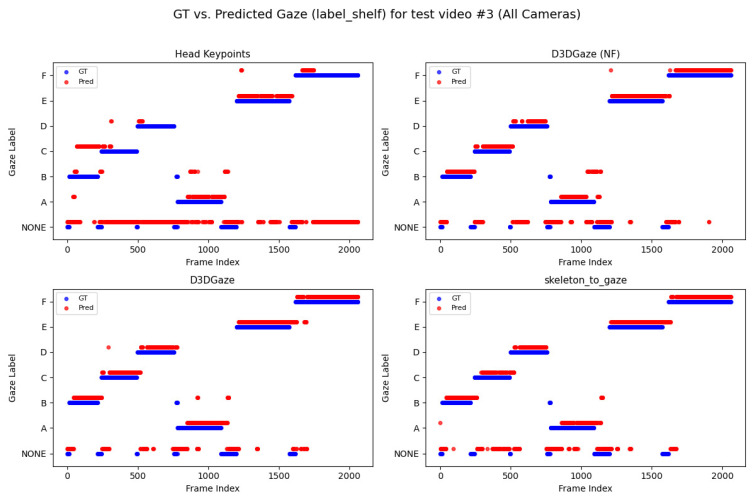
Comparison of ground truth (red) and predicted (blue) gaze directions for the label shelf case on test video 3.

**Figure 23:**
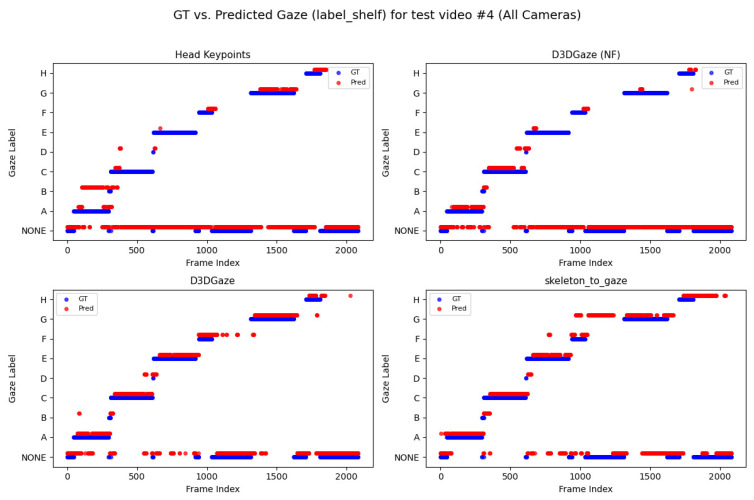
Comparison of ground truth (red) and predicted (blue) gaze directions for the label shelf case on test video 4.

**Figure 24:**
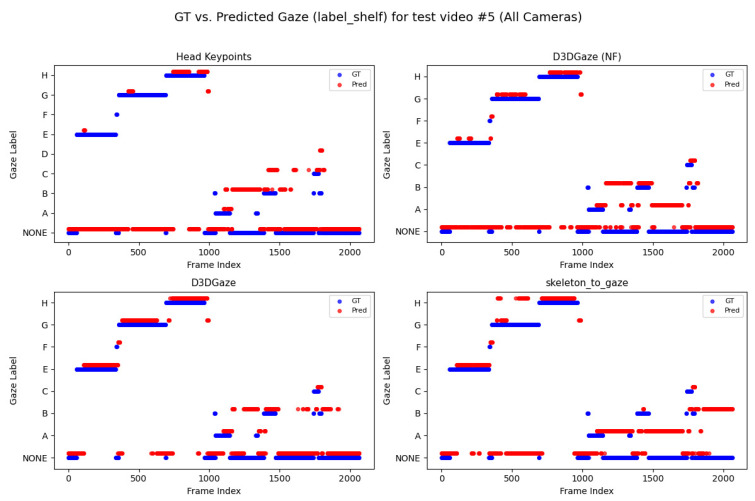
Comparison of ground truth (red) and predicted (blue) gaze directions for the label shelf case on test video 5.

Looking across Figure 20, Figure 21, Figure 22, Figure 23 and Figure 24 for test video nos. 1 through 5, we observe a generally consistent pattern in how each method (head keypoints, D3DGaze(NF), D3DGaze, skeleton-to-gaze) tracks the subject’s gaze toward the label shelf case. In each subplot, red markers represent ground truth annotations, and blue markers represent the method’s predictions. A clear takeaway is that the two D3DGaze methods, particularly the fine-tuned D3DGaze method, tend to align more closely with the ground truth, with fewer abrupt jumps and a tighter clustering of predicted points around the correct gaze label. By contrast, head keypoints and skeleton-to-gaze often exhibit greater deviations in certain segments, likely due to inferior performance on the gaze estimation task.

Notably, the differences among the methods become more pronounced in frames where the subject’s head orientation changes only slightly or when the subject’s body is positioned in a way that does not reliably indicate eye direction. For example, in many of these figures, you can spot periods (e.g., from frame ∼500 to ∼1000 or ∼1500 to ∼2000) where the ground truth shifts among multiple labels (NONE, F, G, H), but head keypoints and skeleton-to-gaze lag behind or jump incorrectly between labels. This discrepancy underscores the difficulty of inferring gaze purely from coarse pose or skeletal cues, especially if the subject’s eyes can move independently of their head or torso.

Between the two D3DGaze plots, D3DGaze(NF) and the fine-tuned D3DGaze, there is a recurring trend where D3DGaze(NF) occasionally displays slightly more scattered predictions (more “blue dots” landing away from the red ground truth clusters). The fine-tuned D3DGaze generally appears smoother and more consistently aligned with the red markers.

While 3D gaze estimation methods like D3DGaze excel in capturing subtle eye movements, the proposed skeleton-to-gaze method offers a compelling alternative by leveraging comprehensive body pose information to infer gaze direction. This method harnesses the rich dynamics of the entire skeleton, making it particularly effective in scenarios characterized by pronounced head and body movements. The skeleton-to-gaze method is well-suited for environments where large-scale movements are prevalent. It can be deployed as long as 3D skeleton data are available, regardless of background changes. In practical applications such as human–robot interaction, attention monitoring, or behavioral analysis, the skeleton-to-gaze approach not only provides robust performance on its own but also complements more sophisticated methods, enhancing overall system reliability across diverse viewing conditions and subject behaviors.

**Figure 25:**
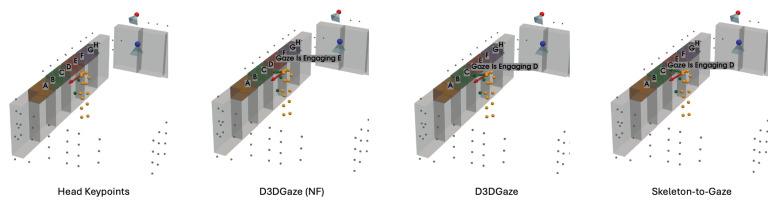
Example frame from test video 1 at frame 900 illustrating gaze-object engagement detection using different gaze estimation methods. Red arrows are ground-truth gaze vectors; green arrows are model gaze estimates; blue arrows are head-keypoint gaze estimates.

**Figure 26:**
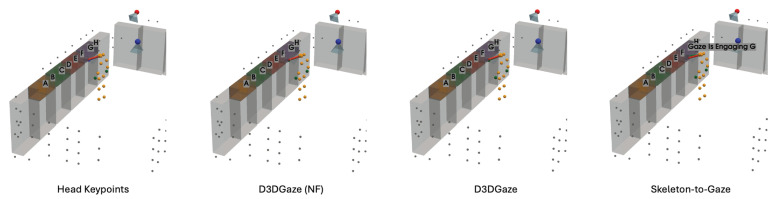
Example frame from test video 2 at frame 750 illustrating gaze-object engagement detection using different gaze estimation methods. Red arrows are ground-truth gaze vectors; green arrows are model gaze estimates; blue arrows are head-keypoint gaze estimates.

**Figure 27:**
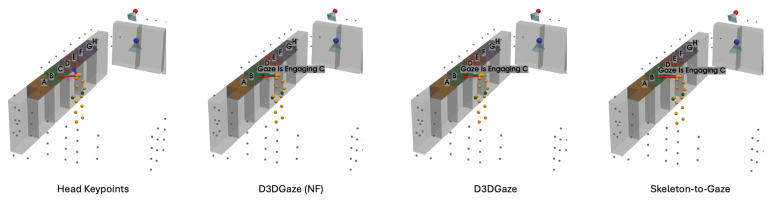
Example frame from test video 3 at frame 400 illustrating gaze-object engagement detection using different gaze estimation methods. Red arrows are ground-truth gaze vectors; green arrows are model gaze estimates; blue arrows are head-keypoint gaze estimates.

**Figure 28:**
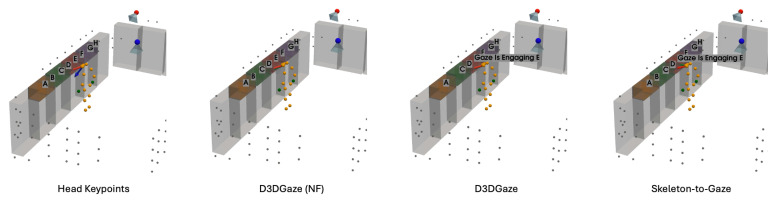
Example frame from test video 4 at frame 750 illustrating gaze-object engagement detection using different gaze estimation methods. Red arrows are ground-truth gaze vectors; green arrows are model gaze estimates; blue arrows are head-keypoint gaze estimates.

**Figure 29:**
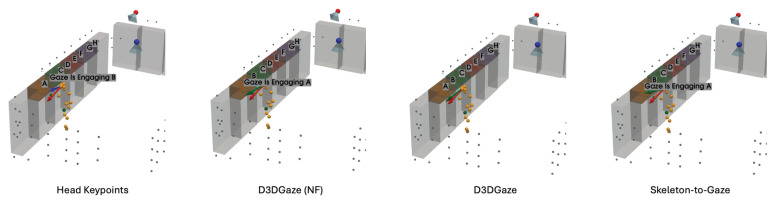
Example frame from test video 5 at frame 1530 illustrating gaze-object engagement detection using different gaze estimation methods. Red arrows are ground-truth gaze vectors; green arrows are model gaze estimates; blue arrows are head-keypoint gaze estimates.

To explain the frequent misclassifications by the head keypoints method and the false-positive gaze-object engagement detections produced by the D3DGaze(NF), D3DGaze, and skeleton-to-gaze models as presented by the confusion matrices in Figure 19 and the graphs in Figure 20, Figure 21, Figure 22, Figure 23 and Figure 24. Figure 25, Figure 26, Figure 27, Figure 28 and Figure 29 show example frames illustrating gaze-object engagement detection using different gaze estimation approaches. The red arrows indicate the ground truth gaze vectors, the green arrows represent the estimated gaze vectors from the gaze estimation models, and the blue arrows show the estimated gaze vectors from the head keypoints approach.

Figure 25 corresponds to test video 1 at frame 900, where the subject is reading the product label after picking it from the shelf. In this scenario, the gaze estimation models tend to inaccurately estimate the gaze direction, leading to false-positive detection, whereas the head keypoints method can handle this situation because the gaze vector is directly estimated from the head pose.

Next, Figure 26 corresponds to test video 2 at frame 750, where the estimated gaze vectors point too far downward relative to the shelf containing the product, resulting in the engagement detection being missed. Additionally, Figure 27 and Figure 28 correspond to test video 3 at frame 400 and test video 4 at frame 750, respectively. These figures showcase scenarios in which the head keypoints method tends to fail because head pose information alone is insufficient for the gaze estimation task.

Lastly, Figure 29 corresponds to test video 5 at frame 1530. This example illustrates another scenario in which the subject checks the product after picking it up from the shelf; this is a challenging situation for accurately estimating gaze vectors, which in turn leads to false-positive gaze-object engagement detection.

### 4.6. Analysis of Hand-Gaze Aggregation

**Figure 30:**
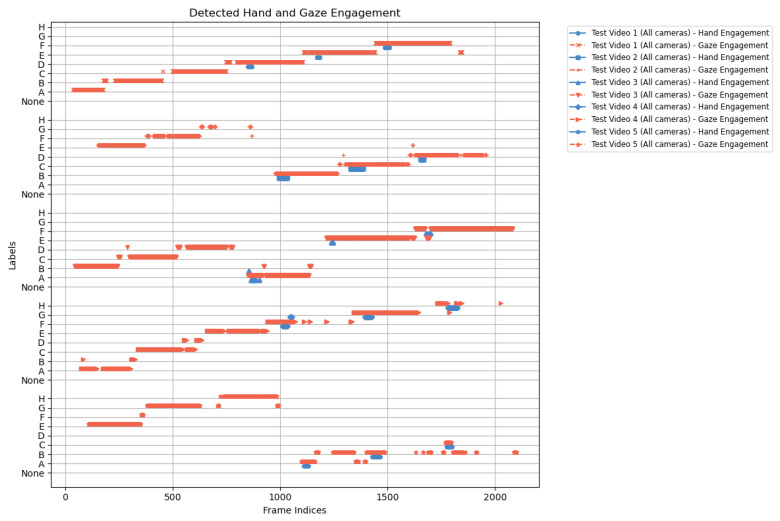
Predicted hand-object and gaze-object engagements across five test videos, illustrating brief physical interactions versus extended visual attention.

Finally, we investigate how combining hand-object and gaze-object engagement signals can offer deeper insights into customer behavior. Figure 30 reveals several key insights into customer behavior by placing the predicted hand-object engagement side by side with the gaze-object engagement across five test videos. Although hand-object engagements appear short, primarily due to the detection method relying on the precise overlap of the hand and shelf in 3D space, these brief moments can pinpoint the exact timing of when a customer physically interacts with a product. In contrast, the gaze-object engagement signals tend to last longer, suggesting that customers often fixate visually on a product for extended periods, even if they do not immediately handle it. This combination of metrics indicates that while a fleeting hand contact might be a strong indicator of purchase intent, prolonged gaze may capture the initial interest or consideration phase. Ultimately, such insights can help retailers optimize product placements and store layouts to cater to both the visual attention and the physical interaction patterns of shoppers, thereby enhancing the overall customer experience and informing targeted marketing strategies.

## 5. Discussion

In this section, we discuss the insights derived from our evaluation of 3D keypoint reconstruction, hand-object detection, and gaze-object estimation. Our analysis examines both the strengths and limitations of the proposed methods in capturing customer engagement dynamics. By interpreting quantitative metrics and qualitative observations, we highlight how precise physical interactions and sustained visual attention contribute to a deeper understanding of consumer behavior. Furthermore, we explore potential avenues for future improvements to enhance system robustness and reliability in real-world retail scenarios.

### 5.1. Hand-Object Detection Strengths

Our hand-object detection method, which estimates engagement based on the precise 3D overlap of the hand with the shelf, offers high temporal precision in capturing physical interactions. Despite the relatively short duration of these detections, owing to the strict overlap requirement, this approach is highly effective at pinpointing the exact moments when a customer physically touches a product. The method’s ability to deliver low error metrics, as indicated by our evaluation results, demonstrates its robustness and suitability for video analysis. Such precise detection is instrumental for applications like dynamic product placement and inventory management, where understanding the exact timing of physical interactions is crucial.

### 5.2. Gaze-Object Detection Strengths

In contrast, our gaze-object detection approach captures the prolonged visual attention of customers by measuring the overlap between gaze vectors and the shelf’s voxel volumes. The extended duration of gaze engagements provides valuable insights into the initial phases of consumer interest and product consideration. This method is particularly beneficial for identifying products that attract attention, even when they are not physically interacted with, thus offering an additional dimension to customer behavior analysis. The ability to track sustained gaze patterns can help retailers optimize product placements and develop targeted merchandising strategies that align with consumer visual engagement.

### 5.3. Advantages of the Proposed Skeleton-to-Gaze Model

Our proposed skeleton-to-gaze model leverages comprehensive body pose information rather than relying solely on head poses or images. By incorporating full skeletal cues via a Transformer-based encoder, the model captures subtle variations in gaze direction accurately, even during rapid movements or complex poses. The competitive performance of this approach, as demonstrated in our experiments, underscores its robustness in diverse scenarios. Not only does it provide a reliable estimation of where a customer is looking, but it also complements more advanced 3D gaze estimation methods. Furthermore, because it relies solely on 3D skeletons, the model is inherently invariant to changes in backgrounds and lighting conditions, making it especially effective in cluttered or dynamically changing environments. With the availability of a large-scale dataset to train the model, it becomes particularly valuable in applications that require robust behavioral analysis and human–robot interaction.

### 5.4. Comparison with Existing Gaze Estimation Methods

Although many prior gaze estimation models exist, we did not include them in our evaluation due to fundamental differences in data requirements and scene assumptions. Most existing methods rely on close-up, high-resolution facial images or eye crops, which are not available in our multi-camera retail setup. Others require semantic object annotations or operate only in constrained environments. Methods based solely on head orientation are also not directly comparable, as they do not produce 3D gaze vectors aligned with our ground truth. Our proposed skeleton-based framework is specifically designed to operate in cluttered, occluded, and privacy-sensitive retail scenarios, where appearance-based models are less applicable.

### 5.5. Limitations of Hand-Object Detection

A notable limitation of the hand-object detection method is that, while it accurately determines the timing of physical interactions, it does not offer granular information regarding which specific product is being picked up or whether it is subsequently returned to the shelf. This lack of detailed insight restricts the understanding of finer behavioral nuances, such as product comparison or decision hesitation. To address this shortcoming, additional close-up cameras or higher-resolution imaging near the shelves could provide more detailed views, enabling better identification of the products in question and facilitating a more comprehensive analysis of customer interactions.

### 5.6. Limitations of Gaze-Object Detection

Similarly, the gaze-object detection method faces challenges due to its reliance on computing engagement based on the overlap between the gaze vector and the shelf’s voxel volumes. When a product is not positioned on the shelf, such as when it is held by the customer, the overlap is reduced, leading to lower detection metrics. Furthermore, image-based approaches like D3DGaze can be sensitive to environmental factors, such as lighting and occlusion, which may affect the accuracy of gaze estimation. These limitations indicate that improvements in sensor fusion or the development of more robust gaze-tracking algorithms are necessary for more consistent performance.

### 5.7. Future Work and Potential Improvements

Future work should aim to overcome these limitations by integrating additional sensor modalities and optimizing camera placements. For hand-object detection, incorporating close-up cameras near shelves could capture finer product details and improve the identification of specific items being handled. For gaze-object detection, employing multi-modal fusion, combining visual data with depth or infrared sensors, could mitigate issues caused by occlusion or variable lighting. Moreover, advanced deep learning architectures such as attention-based models or hybrid systems that merge hand and gaze information may further enhance the reliability and accuracy of engagement detection. Validating these improvements in real-world, large-scale retail environments will be essential for refining these methods and fully leveraging their potential for customer behavior analysis. Additionally, lightweight models for real-time applications are necessary.

## 6. Conclusions

In this paper, we presented a comprehensive system for product engagement detection that integrates multi-camera 3D skeleton reconstruction with gaze estimation. By leveraging a 360-degree top-view fisheye camera alongside two perspective cameras, our framework effectively captures both hand-object and gaze-object interactions in a retail environment. A robust AprilTag-based calibration process enabled the precise reconstruction of the retail space and accurate extraction of 3D human keypoints, forming the basis for both hand tracking and gaze estimation.

Central to our approach is our proposed Transformer-based skeleton encoder for gaze estimation, which leverages raw 3D skeletal cues to filter out extraneous visual details. Although its performance is comparable to existing image-based methods, this novel encoder offers a distinct advantage by being less sensitive to background variations and lighting changes, addressing key challenges of conventional approaches. This achievement demonstrates that our system effectively transforms the theoretical insights presented in the introduction into a practical, data-driven framework for customer behavior analysis.

Overall, our work provides valuable insights into customer interactions that can inform product placement, merchandising, and marketing strategies. Future research will focus on further refining these methods and extending the framework to diverse retail scenarios.

## Figures and Tables

**Table 1:** Numerical summary of the dataset used for evaluating reconstructed 3D keypoints. For each action, the table lists the number of videos recorded from four modalities (Cam1, Cam2, fisheye, and MoCap), along with the number of synchronized frames and total 3D keypoints captured by the motion capture system.

Actions	Side-View Cam 1	Side-View Cam 2	Fisheye	MoCap	#Sync Frames	#Keypoints
Pick Items	15 videos	15 videos	15 videos	15 videos	2471	96,369
Return Items	15 videos	15 videos	15 videos	15 videos	2385	93,015
Read Labels	15 videos	15 videos	15 videos	15 videos	3826	149,214
Put in Basket	15 videos	15 videos	15 videos	15 videos	2225	86,775
Check Watch	15 videos	15 videos	15 videos	15 videos	3497	136,383
Total	75 videos	75 videos	75 videos	75 videos	14,404	561,756

**Table 2:** Overview of the fine-tuning dataset. This table presents the number of videos and frames in the training, validation, and test sets for both perspective and fisheye views, summarizing the key details of the dataset used to fine-tune the gaze estimation model.

Category	Perspective View	Fisheye View
No. of videos	476 videos	104 videos
Train set	350 videos, 1,684,922 frames	64 videos, 223,779 frames
Validation set	60 videos, 316,092 frames	20 videos, 85,715 frames
Test set	66 videos, 335,124 frames	20 videos, 76,052 frames

**Table 3:** Summary of gaze and hand interaction cases. This table outlines the different scenarios in which subjects engaged with products, either by looking at them or picking them from the shelves.

Case	Gaze-Object	Hand-Object
1	A, B, C	D, E, F
2	E, F, G	B, C, D
3	B, C, D	A, E, F
4	A, C, E	F, G, H
5	E, G, H	A, B, C

**Table 4:** Detailed summary of the product engagement dataset. This table presents the number of videos and frames captured for the experiment, along with relevant metadata for each recording condition.

	Perspective View	Fisheye View
No. of videos	52 videos	26 videos
Test set	52 videos, 92,514 frames	26 videos, 46,257 frames

**Table 5:** Evaluation of a 3D reconstruction of keypoints.

Cameras	MSE (m^2^)	RMSE (mm)
All Cameras	0.000524	22.89
Cam1 & Fisheye	0.000576	24.01
Cam2 & Fisheye	0.003501	59.17
Cam1 & Cam2	$9.90\times 10^{-17}$	$9.95\times 10^{-6}$

**Table 6:** Ablation study and inference time comparison of skeleton-to-gaze and D3DGaze models on the fine-tuning dataset. The table reports the 3D mean angular error (MAE) and inference speed in frames per second (FPS). Variants of the skeleton-to-gaze model are evaluated by removing the head or body loss component to assess their impact on performance.

Method	3D MAE (°)	Inference Time (FPS)
D3DGaze (NF)	34.19	1476.51
D3DGaze	31.05	1482.31
Skeleton-to-gaze (ours)	**29.83**	**2401.32**
Skeleton-to-gaze (w/o head loss)	30.67	2460.25
Skeleton-to-gaze (w/o body loss)	31.74	2469.59

**Table 7:** Evaluation of gaze estimation methods on the product engagement dataset.

Method	3D MAE (°)	No. of Parameters
Head keypoints	35.50	-
Skeleton-to-gaze (ours)	29.13	88.07 M
D3DGaze (NF)	**23.31**	9.465 M
D3DGaze	25.33	9.465 M

**Table 8:** Evaluation metrics (accuracy, balanced accuracy, and weighted F1) for hand-object engagement detection on 2440 “label shelf” frames. The “Cameras Used” column reflects various configurations.

Cameras Used	Ground Truth	No. Frames	Accuracy	Balanced Accuracy	Weighted F1
All Cameras	Label Shelf	2440	0.7910	0.8126	0.8724
All Cameras *	Label Shelf	2440	**0.8385**	**0.8550**	**0.8994**
Cam1,Cam2	Label Shelf	2440	0.5988	0.6055	0.6794
Cam1,Cam2 *	Label Shelf	2440	0.6824	0.6859	0.7196
FisheyeCam2	Label Shelf	2440	0.5811	0.6010	0.6691
FisheyeCam2 *	Label Shelf	2440	0.6197	0.6351	0.6970
FisheyeCam1	Label Shelf	2,440	0.7004	0.7275	0.8003
FisheyeCam1 *	Label Shelf	2440	0.7795	0.7946	0.8488

* Distance compensation of 15 cm applied for the wrist skeleton and hand location.

**Table 9:** Evaluation of gaze-object engagement detection on the product engagement dataset.

Method	Cameras Used	Ground Truth	No. Frames	Accuracy	Balanced Accuracy	Weighted F1
Head keypoints	All Cameras	Label Shelf	27,702	0.1754	0.1825	0.2692
Head keypoints	All Cameras	Label Product	11,927	0.1836	0.2186	0.2681
Head keypoints	All Cameras	Label Both	35,812	0.1725	0.1458	0.2627
D3DGaze (NF)	All Cameras	Label Shelf	27,702	0.4799	0.4668	0.6225
D3DGaze (NF)	All Cameras	Label Product	11,927	0.3407	0.3419	0.4520
D3DGaze (NF)	All Cameras	Label Both	35,812	0.4361	0.3375	0.5718
D3DGaze	All Cameras	Label Shelf	27,702	**0.5245**	**0.5167**	**0.6653**
D3DGaze	All Cameras	Label Product	11,927	0.4213	0.4536	0.5464
D3DGaze	All Cameras	Label Both	35,812	0.4922	0.3871	0.6326
Skeleton-to-gaze	All Cameras	Label Shelf	27,702	0.5047	0.5019	0.6410
Skeleton-to-gaze	All Cameras	Label Product	11,927	0.4719	0.4846	0.5858
Skeleton-to-gaze	All Cameras	Label Both	35,812	0.5023	0.3989	0.6324

## Data Availability

The datasets presented in this article are not readily available, as they are part of an ongoing study. Requests for access to the datasets should be directed to tanonwong.matus.q5@dc.tohoku.ac.jp.
